# A facile strategy for tuning the density of surface-grafted biomolecules for melt extrusion-based additive manufacturing applications

**DOI:** 10.1007/s42242-024-00286-2

**Published:** 2024-05-20

**Authors:** I. A. O. Beeren, G. Dos Santos, P. J. Dijkstra, C. Mota, J. Bauer, H. Ferreira, Rui L. Reis, N. Neves, S. Camarero-Espinosa, M. B. Baker, L. Moroni

**Affiliations:** 1 Department of Complex Tissue Regeneration, MERLN Institute for Technology-Inspired Regenerative Medicine, Maastricht University, 6229 ER Maastricht, The Netherlands; 2 3B’s Research Group, I3Bs – Research Institute on Biomaterials, Biodegradables and Biomimetics, Headquarters of the European Institute of Excellence on Tissue Engineering and Regenerative Medicine, University of Minho, AvePark, Zona Industrial da Gandra, 4805-017 Barco, Guimarães, Portugal; 3 ICVS/3B’s-PT Government Associate Laboratory, 4806-909 Braga/Guimarães, Portugal; 4 POLYMAT, University of the Basque Country UPV/EHU, 20018 Donostia/San Sebastián, Spain; 5 IKERBASQUE, Basque Foundation for Science, 48009 Bilbao, Spain

**Keywords:** Additive manufacturing, Blending, Surface functionalization, Surface density, Click chemistry, Human mesenchymal stromal cell (hMSC) differentiation

## Abstract

Melt extrusion-based additive manufacturing (ME-AM) is a promising technique to fabricate porous scaffolds for tissue engineering applications. However, most synthetic semicrystalline polymers do not possess the intrinsic biological activity required to control cell fate. Grafting of biomolecules on polymeric surfaces of AM scaffolds enhances the bioactivity of a construct; however, there are limited strategies available to control the surface density. Here, we report a strategy to tune the surface density of bioactive groups by blending a low molecular weight poly(ε-caprolactone)_5k_ (PCL_5k_) containing orthogonally reactive azide groups with an unfunctionalized high molecular weight PCL_75k_ at different ratios. Stable porous three-dimensional (3D) scaffolds were then fabricated using a high weight percentage (75 wt.%) of the low molecular weight PCL_5k_. As a proof-of-concept test, we prepared films of three different mass ratios of low and high molecular weight polymers with a thermopress and reacted with an alkynated fluorescent model compound on the surface, yielding a density of 201–561 pmol/cm^2^. Subsequently, a bone morphogenetic protein 2 (BMP-2)-derived peptide was grafted onto the films comprising different blend compositions, and the effect of peptide surface density on the osteogenic differentiation of human mesenchymal stromal cells (hMSCs) was assessed. After two weeks of culturing in a basic medium, cells expressed higher levels of BMP receptor II (BMPRII) on films with the conjugated peptide. In addition, we found that alkaline phosphatase activity was only significantly enhanced on films containing the highest peptide density (i.e., 561 pmol/cm^2^), indicating the importance of the surface density. Taken together, these results emphasize that the density of surface peptides on cell differentiation must be considered at the cell-material interface. Moreover, we have presented a viable strategy for ME-AM community that desires to tune the bulk and surface functionality via blending of (modified) polymers. Furthermore, the use of alkyne–azide “click” chemistry enables spatial control over bioconjugation of many tissue-specific moieties, making this approach a versatile strategy for tissue engineering applications.

**Graphic abstract:**

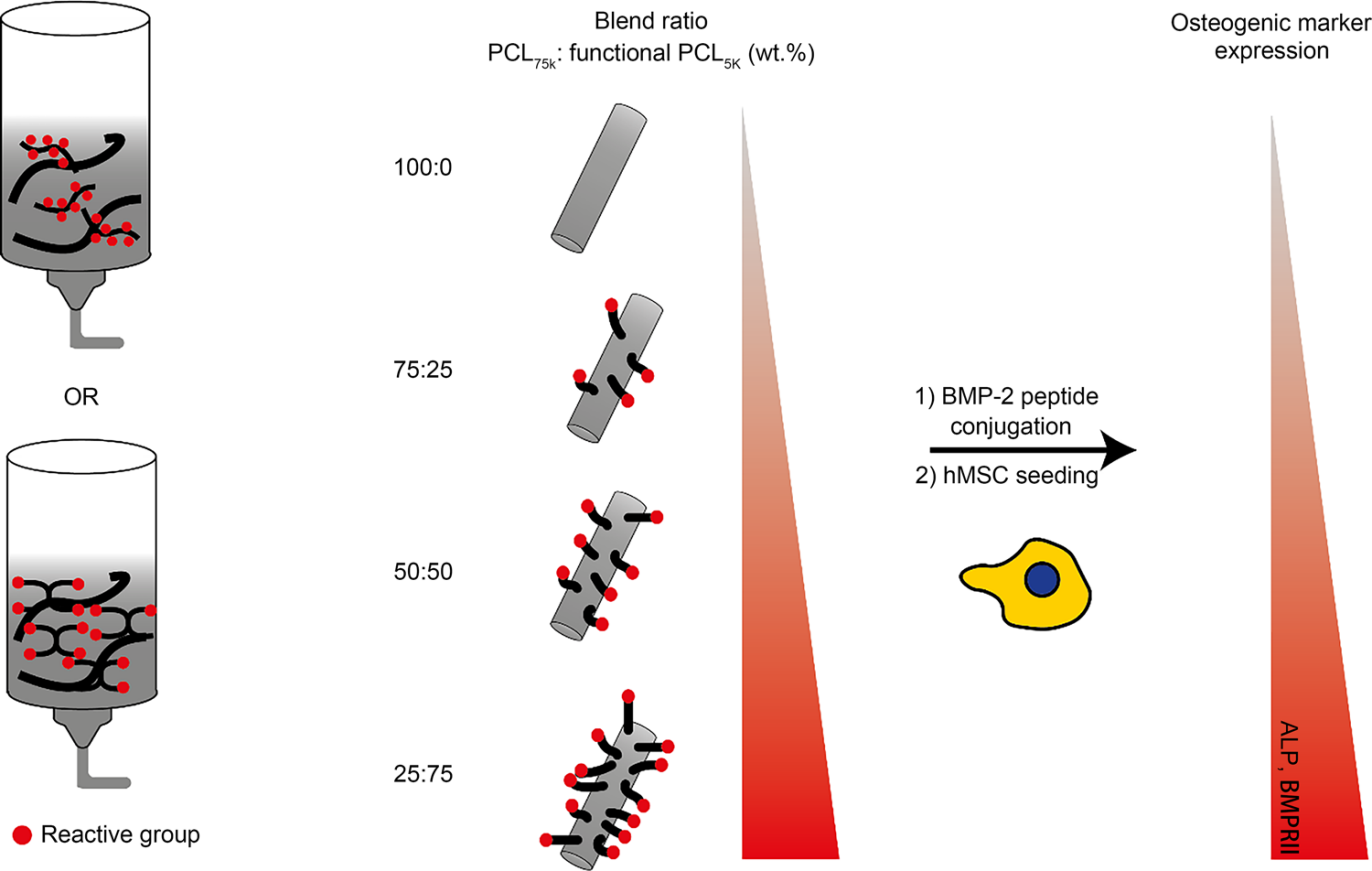

**Supplementary Information:**

The online version contains supplementary material available at 10.1007/s42242-024-00286-2.

## Introduction

Melt extrusion-based additive manufacturing (ME-AM) techniques, such as fused deposition modeling or three-dimensional (3D) fiber deposition, are promising scaffold fabrication technologies in the field of tissue engineering. This is because ME-AM enables the fabrication of 3D scaffolds with precise control over their shape, pore size, and interconnectivity [1–4]. In addition, the architecture of the scaffold can be varied to modulate the scaffold’s mechanical properties [5–7] and seeding efficiency [8]. Generally, semicrystalline polymers are used to prepare ME-AM scaffolds for tissue engineering applications, such as poly(ε-caprolactone) (PCL) [9], polylactic acid [10], poly(ester-urethane)s [11, 12], and poly(ethylene oxide terephthalate)/poly(butylene terephthalate) [5, 13]. These polymers, which have melting temperatures that are well above body temperature, provide stable scaffolds after melt extrusion at ambient/body temperatures [14]. The intrinsic properties of these materials of choice, such as substrate stiffness and stress relaxation, influence the behavior of seeded cells [15–17]. However, from a tissue engineering point of view, these synthetic materials do not possess inherent bioactivity required to control the cell fate. To address this limitation, various strategies to biofunctionalize the surfaces of scaffolds with proteins or peptides have been applied.

However, since diseases often affect tissue interfaces, a functionalizaton strategy should ideally permit a heterogenous distribution of the bioactivity across scaffolds. For example, in the osteochondral unit, where ME-AM scaffolds are widely applied due to their inherent mechanical properties [18–20], osteoarthritis affects both cartilage and subchondral bone homeostasis [21]. Thus, when a scaffold is designed to assist regeneration of this affected interface, the presentation of chondrogenic and osteogenic factors is required on opposite side of the scaffold.

For example, thermoplastic polymers have been functionalized with different biomolecules, prior to ME-AM, and consecutively deposited to form a multilayered scaffold. In particular, Guo et al. modified the end groups of PCL with cartilage- or bone-specific peptides and showed that chondrogenic and osteogenic extracellular matrix deposition was promoted in rat mesenchymal stromal cells (MSCs) on scaffolds [22]. Similarly, Camacho et al. prefunctionalized low molecular weight PCL_14k_ with hyaluronic acid-binding or mineralization peptides and ensured printability via implementation of a blending strategy using high molecular weight PCLs. After the fabrication of a bilayered construct, seeded human mesenchymal stromal cells (hMSCs) exhibited higher expression levels of osteogenic markers [23]. One drawback of biofunctionalization prior to manufacturing is the possible loss of bioactivity after ME-AM as many biomolecules are sensitive to high temperatures and/or shear forces.

As an alternative method, biomolecules can be grafted onto the surface of AM scaffolds post-fabrication. For example, previous studies have used aminolysis reactions or plasma treatment to functionalize the surface of scaffolds, typically in homogenous fashion [24–26]. To circumvent this homogenous distribution, Di Luca et al. used capillary forces to introduce differentiation-inducing proteins in a gradual fashion via carbodiimide-coupling chemistry. However, the effect of the proteins on hMSC differentiation was limited, possibly due to low ligand density [27]. Carbodiimide-coupling chemistry is highly dependent on the carboxylic acid activation step and reactant concentration, which typically results in a low reaction efficiency during surface modifications. Moreover, the presence of amine and carboxylic acid functional groups in many proteins and peptides poses limitations to the spatially controlled grafting of multiple biomolecules.

In the past decades, bio-orthogonal “click” chemistry has emerged as a versatile tool for enabling fast, specific, and efficient reactions of a wide variety of molecules that contain orthogonally reactive functional groups. Click chemistry has also been used to post-functionalize AM fibers. For example, Li et al. applied copper(I)-catalyzed alkyne–azide cycloaddition (CuAAC) chemistry to anchor osteogenesis-inducing peptides on AM fibers of poly(ester urea). Grafting of these peptides promoted osteogenic differentiation of hMSCs [28]. In a recent study from our group, CuAAC or thiol–Michael-addition chemistry was used to attach chondrogenic- or osteogenic-inducing peptides, respectively, onto scaffolds comprising terminally functionalized PCLs. This modification had a small effect on chondrogenesis and no effect on osteogenesis on seeded hMSCs [29]. However, when the spatial distribution across of the orthognally reactive groups can be controlled across the scaffolds, this type of chemistry permits heterogenous grafting of bioactive molecules.

Although the installation of orthogonally reactive groups on the scaffold surface appears a promising strategy for (heterogenous) conjugation of biomolecules, a positive biological effect has not always been reported. We hypothesized that the absence of osteogenesis was related to a too low concentration of peptide on the surface of a scaffold [29]. In general, proteins and peptides are known to exhibit a concentration-dependent effect on stem cell differentiation [30–32]. Moreover, in vivo, too low or too high concentrations have been shown to cause no or adverse effect on physiological processes, respectively [33]. Notably, to the best of our knowledge, no study has yet been able to tune the density of orthogonally reactive groups on the surface of scaffolds prepared via ME-AM, which may enable the tuning of the amount of grafted biomolecule.

**Fig. 1:**
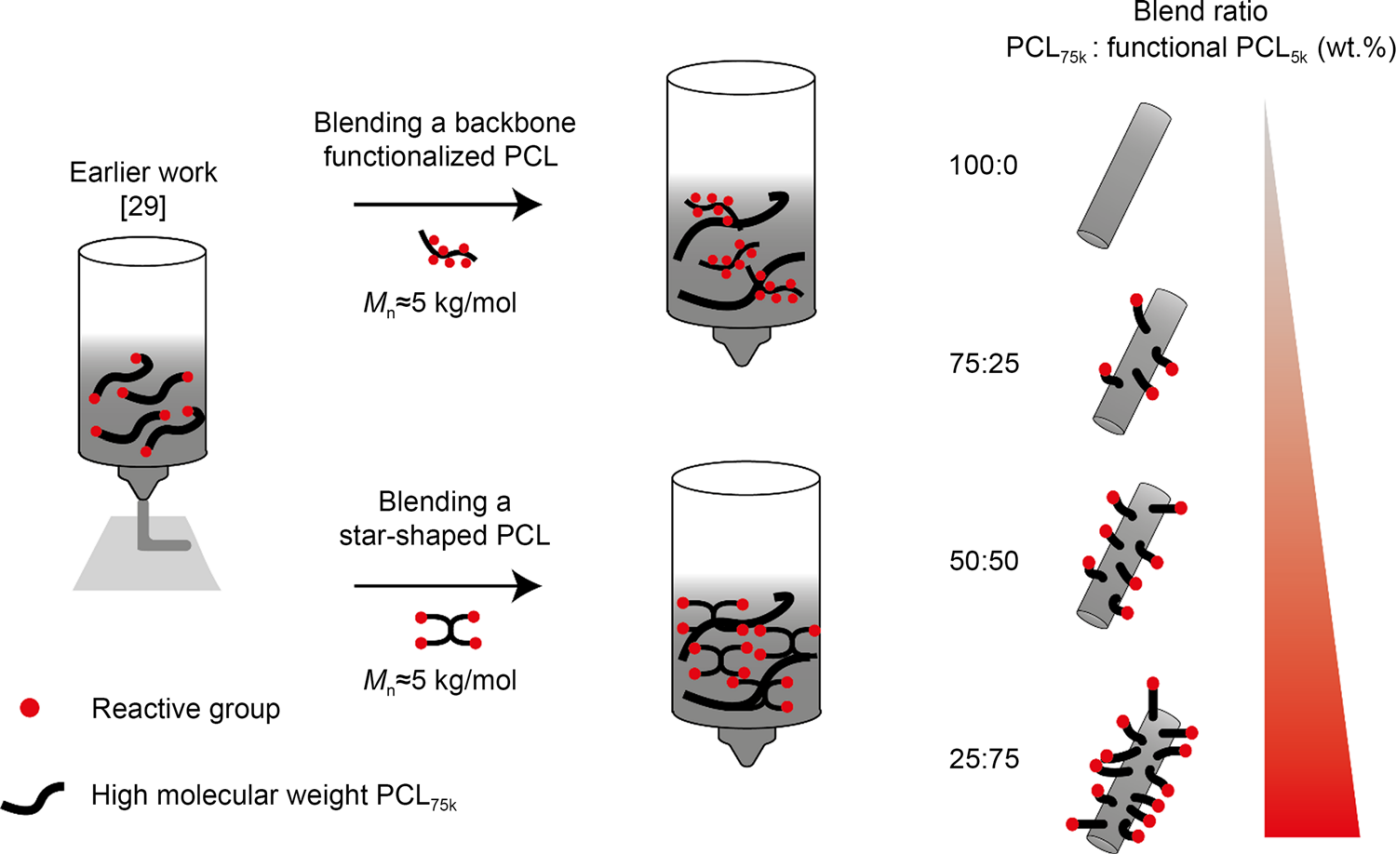
Schematic overview of this study. In an earlier work, the end groups of high molecular weight PCL polymers were modified to introduce biomolecules on the surface [29]. Here, a blending strategy using low and high molecular weight PCLs was applied to enhance the surface density of reactive groups, such as an azide or maleimide groups (left). In addition, using different weight ratios of the indicated polymers enabled the tuning of the availability of addressable groups on the scaffold surface (right). PCL: poly(ε-caprolactone)

In this study, we explored a facile strategy to enhance and tune the surface availability of orthogonal reactive groups on PCL scaffolds (Fig. 1). To do so, we created different blend formulations of low molecular weight PCL (linear or star-shaped) comprising addressable groups with a high molecular weight PCL. Next, we evaluated the stability of these blends when subjected to high temperatures and shear forces during printing. Subsequently, the blends were printed and the shape stability of ME-AM scaffolds and their mechanical properties were evaluated. Instead of traditional linear low molecular weight PCL derivatives comprising two addressable end groups, we have, to the best of our knowledge, for the first time used linear polymers with the addressable group in the backbone and starshaped polymers with addressable end groups in ME-AM applications. To illustrate the ability to tune the surface density of the addressable group via blending, we used a fluorescent model compound containing complementary reactive groups. A bone morphogenetic protein 2 (BMP-2) derived “knuckle” epitope was then selected for surface coupling with blends containing azide groups due to its potential to upregulate bone-specific markers in progenitor cells [34, 35]. Finally, the effect of peptide surface density on the osteogenic differentiation of hMSCs was assessed.

## Materials and methods

### Materials

All chemicals were purchased from Sigma-Aldrich (Saint Louis, Missouri, USA) and used as received, unless stated otherwise. Star-PCL_5k_ was kindly provided by Corbion (The Netherlands). ε-caprolactone (εCL, TCI Chemicals, Zwijndrecht, Belgium) was dried over CaH_2_, distilled, and stored over molecular sieves (3 Å; 1 Å=1×10^−10^ m). Anhydrous toluene and benzyl alcohol were dried over molecular sieves (3 Å). Peptide (sequence: L-propargylglycine-GKIPKASSVPTELSAISTLY) was purchased from Synpeptide Co., Ltd. (Shanghai, China).

### Synthesis

#### Poly(αClεCL-*co*-εCL)

The copolymerization of α-chloro-ε-caprolactone (αClεCL) and εCL was performed according to a previously reported procedure, with slight modifications [36]. To determine the effect of different drying methods prior to copolymerization, αClεCL was either used as received or dried according to several methods (Table S1 in Supplementary Information). In a typical reaction, αClεCL (0.72 g, 4.85 mmol, 50 equivalents) and benzyl alcohol (1.05×10^−4^ g, 9.7×10^−2^ mmol, 1.0 equivalent) were added to an oven-dried Schlenk flask under a dry N_2_ atmosphere. Subsequently, εCL (4.6 mL, 43.7 mmol, 450 equivalents) and anhydrous toluene (3.5 mL) were added to the flask. Finally, stannous octoate (4.91 mg, 1.2×10^−2^ mmol, 0.125 equivalents) was added to the flask; the stock solution of stannous octoate was freshly prepared in anhydrous toluene. The reaction mixture was then freeze-thawed 3–5 times to degas the solution. The reaction mixture was stirred at 120 °C for 27 h under a dry N_2_ atmosphere. Once cooled in ambient environment, the reaction mixture was precipitated in methanol and the product was collected as a white solid that was then dried in vacuo. Yield: 1.75 g, 36%. ^1^H NMR (700 MHz, CDCl_3_): δ 1.38 (m, 74H), 1.64 (m, 159H), 1.95 (m, 5H), 2.04 (m, 5H), 2.31 (t, 72H, *J*=7.5 Hz), 3.65 (dt, 2H), 4.06 (t, 20H, *J*=7.4 Hz), 4.17 (m, 9H), 4.26 (m, 4H), 5.12 (s, 0.2H), 7.35 (m, 0.4H). GPC: see Table S1 (Supplementary Information). Melting point (*T*_m_)=44.3 °C.

#### Poly(αN_3_εCL-*co*-εCL)

In a typical reaction, 1.6 g poly(αClεCL-*co*-εCL) (*M*_n_  of approximately 4.8 kg/mol, 1.4 mmol chloride groups, 1.0 equivalent) was dissolved at a concentration of 200 mg/mL in anhydrous *N*,*N*-dimethylformamide (DMF) under a dry N_2_ atmosphere. Subsequently, NaN_3_ (222 mg, 3.4 mmol, 2.5 equivalents) was added, and the reaction was stirred at room temperature (RT) for 18 h. The reaction mixture was then diluted with dichloromethane, filtered, and then precipitated in cold methanol. The obtained polymer was re-dissolved in chloroform (CHCl_3_) and precipitated in methanol. Thereafter, the final product was dried in vacuo*.* Yield: 1.1 g, 69%. ^1^H NMR (700 MHz, CDCl_3_): δ 1.38 (m, 84H), 1.64 (m, 183H), 1.78 (m, 6H), 1.86 (m, 6H), 2.31 (t, 82H, *J*=7.5 Hz), 3.65 (dt, 2H), 3.84 (m, 5H), 4.06 (t, 81H, *J*=6.8 Hz), 4.20 (m, 10H), 5.12 (s, 0.2H), 7.35 (m, 0.6H). GPC: 7.4 kg/mol, Đ=1.06. *T*_m_=46.3 °C.

#### Poly(αClεCL)

The protocol for the homopolymerization of αClεCL can be found in the Supplementary Information.

#### Star-PCL-maleimide (sPCLM)

The end-group conversion of sPCL_5k_ into sPCLM_5k_ was performed according to the procedure described in a previous report [37]; the method is provided in the Supplementary Information.

### Blend preparation

To generate blends, PCL_75k_ and either sPCL_5k_ or poly(αClεCL-*co*-εCL)_5k_ were first dissolved in CHCl_3_ at a weight ratio of 75:25, 50:50, or 25:75 and a final concentration of 100–150 mg/mL. Subsequently, all solutions were stirred overnight. In each case, the solvent was evaporated at RT and finally dried in vacuo.

### Additive manufacturing of scaffolds

Scaffolds were fabricated using a custom-made ME-AM technique [38]. First, a custom LabView program was used to control temperature and pressure during printing. The extrusion screw was operated using a stepper motor equipped with a Trinamic controller board and software. Stainless steel balls of 3 mm in diameter were placed in the inflow valves. The G-code was generated using GESIM software loaded on a bioscaffolder 3.0 device. We manufactured cylindrically shaped scaffolds of a 25G nozzle, a 0–90 pattern, a 550 μm strand distance, a height of 10 layers, and a diameter of 6 mm. The nitrogen pressure, temperature, screw speed, and translation speed were tweaked to obtain a fiber diameter of approximately 300 μm for each scaffold (Table 1). Finally, the fiber diameter of scaffolds (*n*=2) was measured using Fiji software.

**Table 1:** Extrusion parameters for the fabrication of ME-AM scaffolds using different polymeric blends

	Blend composition	Temperature	Pressure	Screw speed	Translational
	(weight ratio)	(°C)	(bar)	(r/min)	speed (mm/s)
PCL_75k_: sPCL_5k_	100:0	170	3.6	12	390
	75:25	160	3.6	15	300
	50:50	150	3.6	15	360
	25:75	100	3.6	12	420
PCL_75k_: poly(αClεCL-*co*-εCL)_5k_	75:25	115	5.9	7.5	306
	50:50	95	3.6	7.5	372
	25:75	80	0.9	12	360

1 bar = 1×10^5^ Pa. ME-AM: melt extrusion-based additive manufacturing

### Mechanical characterization

The mechanical properties were evaluated by uniaxial compression tests using an ElectroForce 3200 Series III mechanical testing machine (TA Instruments, USA). The instrument was controlled using Wint7 software. Samples (*n*=4 for 75:25 and *n*=3 for 25:75 PCL:sPCL_5k_) were first positioned on a standard compression platform, aligned to a 450 kN load cell, and then compressed at a rate of 0.02 mm/s. Prior to each compression test, all samples were subjected to a 5 N pre-load. The Young’s modulus was calculated from the linear slope (*R*^2^>0.99) of recorded stress–strain curves between 4% and 8% strain. The stress (*σ*) was evaluated as the measured force (*F*) divided by the initial cross-sectional area (*A*) of the scaffold. The strain (*ε*) was calculated as the ratio of the variation in height (Δ*h*) of the scaffold to its initial height (*h*). Both yield stress and strain were determined using the 0.2% offset method.

### Film preparation

Thermopressed films were prepared on silicon wafers using a thermopress. For PCL_75k_ samples, heater blocks were heated to 95 °C. Blends containing poly(αN_3_εCL-*co*-εCL) were heated to 85 °C and (manual) pressure was applied for approximately 5 s. Samples (8 mm in diameter) were punched from the film and fixed with o-rings in a well plate.

### Surface grafting of an alkynated fluorescent model compound

Thermopressed films (*n*=3) of PCL_75k_ and blends of 75:25, 50:50, and 25:75 (wt.%) PCL_75k_ to poly(αN_3_εCL-*co*-εCL) were mounted in well plates as described in  Sect. "Film preparation". A reaction mixture containing 1 mmol/L of alkyne MegaStokes dye 673, 0.5 mmol/L of CuSO_4_·5H_2_O, and 5 mmol/L of sodium ascorbate was prepared in distilled H_2_O. Based on the average weight (approximately 22.5, 12.70, and 8.0 mg, respectively) of the film of each composition, the total number of moles of azide was determined for an 8-mm diameter sample and was set to 1.0 equivalent. An 11% degree of azide functionalization of the backbone was used for all calculations. A volume of the reaction mixture containing 0.1 equivalents of dye was then applied to all blended ratios. Additionally, we added 0.25 equivalents to the surface of the 25:75 film. This protocol was repeated, but without the copper or sodium ascorbate. Subsequently, samples were incubated for 17 h at RT. After incubation, the samples were extensively washed with dimethyl sulfoxide (DMSO, 4×), water (3×), and ethanol (2×) before finally being dried overnight at ambient temperature.

Fluorescence emission spectra were recorded on an Agilent Cary Eclipse Fluorescence Spectrophotometer equipped with a multicell holder and a Peltier device. To do so, films were dissolved in chloroform at 13 mg/mL and excited at 550 nm. The emissions between 580 and 800 nm were then recorded. A medium scan speed was applied with a 5 nm excitation slit width and 800 V detector sensitivity. The dye concentration was calculated against a standard curve.

### Surface grafting of an alkynated BMP-2-derived peptide

Thermopressed films of PCL_75k_ as well as blends of 75:25 and 25:75 (wt.%) of PCL_75k_ to poly(αN_3_εCL-*co*-εCL) were punched and sterilized in 70% ethanol. These films were mounted in 48 untreated well plates using o-rings and washed twice with a phosphate-buffered saline (PBS) solution. Stock solutions of CuSO_4_·5H_2_O (1 mg/mL), sodium ascorbate (10 mg/mL), and alkynated BMP-2 peptide (10 mg/mL) were prepared in distilled H_2_O. A reaction mixture comprising 1 mmol/L BMP-2 peptide, 0.5 mmol/L CuSO_4_·5H_2_O, and 5 mmol/L sodium ascorbate in distilled H_2_O was used. Based on the average weight of the films, the total number of moles of azide in the film (8-mm diameter) was determined and set to 1.0 equivalent. For example, for films with mass ratios of 75:25 and 25:75, 408 and 850 μL of the reaction mixtures were incubated, respectively, containing 0.1 equivalents of the alkynated BMP-2 peptide. The reaction was then left for 17 h at RT. Finally, all films were washed four times with PBS prior to cell seeding.

### hMSC culture and seeding

Human bone marrow-derived MSCs were isolated by the Texas A&M Health Science Center from a 22 year old male donor [39]. In brief, mononuclear cells were isolated via centrifugation of aspirated bone marrow. The hMSCs were further expanded and their differentiation potential was verified. Cells (P1) were plated at 1000 cells/cm^2^ in tissue culture flasks, and expanded in alpha minimum essential medium with GlutaMax containing no nucleoside (Gibco). This was supplemented with 10% *v/v* fetal bovine serum at 37 °C and 5% CO_2_ until 70%–80% confluence was reached. Cells were used in experiments at passage 5.

### Evaluation of differentiation markers

#### Alkaline phosphatase (ALP) activity assay

After 7 (*n*=3) and 14 (*n*=4) days, films were washed with PBS and were freeze-thawed three times. Samples were submerged in cell lysis buffer (0.1 mol/L KH_2_PO_4_, 0.1 mol/L K_2_HPO_4_, and 0.1% *v/v* triton-X-100, pH 7.8) for 1 h at RT. Next, a total of 10 μL of the lysate and 40 μL CDP-star^®^ (Chemiluminescent Substrate) were added to a black 96 well plate. After 15 min of incubation, luminescence (*λ*_em_=466 nm) was measured using a spectrophotometer (CLARIOstar^®^, BMG LABTECH, Germany). The remainder of the lysate was used for DNA quantification. Values were normalized to DNA content.

#### DNA quantification

Lysed samples from the ALP (see Sect. “Alkaline phosphatase (ALP) activity assay”) were diluted one-to-one (*v/v*) in a proteinase K lysis buffer (1 mg/mL in Tris/EDTA, pH 7.6). These samples were incubated overnight at 56 °C before being immediately freeze-thawed three times. The amount of DNA was then quantified using a CyQUANT cell proliferation assay kit (Thermo Fisher Scientific, The Netherlands). Briefly, all samples were diluted in a one-to-one volume ratio with the lysis buffer from the kit containing DNase-free RNase A (final dilution = 1:1000 *v/v*) to degrade cellular RNA. After 1 h of incubation, samples were incubated with fluorescent dye (1:200) for 12 min. Fluorescence was measured at *λ*_ex_=480 nm and *λ*_em_=520 nm using a spectrophotometer. Finally, DNA concentrations were determined from a standard curve.

#### Immunofluorescence staining

Samples (*n*=2) were fixed in 4% paraformaldehyde, washed with PBS, and permeabilized for 15 min in a 0.1% *v/v* Triton-X100 solution in PBS. Subsequently, the samples were blocked for 1 h with 3% *v/v* bovine serum albumin and 0.01% *v/v* Triton-X100 in PBS. After washing with PBS, all samples were incubated overnight at 4 °C with rabbit anti-osteocalcin (1:300, Ab93876), anti-BMP receptor II (anti-BMPRII, 1:400, NBP2-37,624), and AlexaFluor Phalloidin-488 (1:100) in PBS. Then, samples were rinsed with 0.3% *v/v* bovine serum albumin and 0.01% Triton-X100 in PBS. Goat anti-rabbit and goat antimouse secondary antibodies were incubated for 30 min at RT (1:200, either conjugated with Alexa Fluor 568 or 647), and shielded from light. After washing with PBS, cell nuclei were counterstained with 4',6-diamidino-2-phenylindole (DAPI, 0.233 μg/mL) for 15 min. Samples were then imaged using an inverted epifluorescence microscope. The background signal was removed via the Fiji version 2.9 software (NIH, USA). During processing, a constant rolling ball radius was maintained for each channel and brightness was slightly adjusted.

### Statistical analysis

All data are presented as mean±standard deviation. Statistical analysis was performed using GraphPad Prism version 9.5.1. After confirming that the data were normally distributed using a Shapiro–Wilk test, we determined the statistical significance by a one-way analysis of variance followed by Tukey’s honestly significant difference multiple comparison tests. In case of non-normal data distribution, a Kruskal–Wallis test and a Dunn’s multiple comparison were performed. Significance of differences is expressed as ^****^p<0.0001, ^***^p<0.001, ^**^p<0.01, and ^*^p<0.05.

## Results and discussion

In a previous study of our group, the functionalization of the surface of a scaffold composed of high molecular weight PCL with terminal azide groups was reported [29]. From a biological perspective, an enhancement of the number of available reactive groups on the surface was desired. To this end, we here applied a strategy based on the blending of low molecular weight PCL derivatives comprising orthogonally addressable groups and high molecular weight PCLs. To modulate the density of functional groups available on the surface, blends containing different amounts of functional low molecular weight PCLs were subsequently prepared (Fig. 1). However, only the terminal hydroxyl groups of linear bifunctional PCL polymers can be functionalized, limiting the possibility of covering a broader (and higher) range of surface densities. Therefore, we set out to synthesize PCL-derived polymers that contain a higher number of reactive groups per chain than linear bifunctionalized PCL polymers: 1) A linear PCL with chloride groups (poly(αClεCL-*co*-εCL)_5k_) and 2) a star-shaped PCL (sPCL_5k_) with four hydroxyl end groups were synthesized. In these polymers, the chloride as well as hydroxyl groups can be efficiently converted into orthogonally reactive azide and maleimide groups at a later stage [40, 41].

In Table 2, we illustrated the theoretical changes in the number of millimoles of reactive groups (i.e., Cl or OH) per gram present in the melt by varying the weight ratio of the low to high molecular weight PCL-derived polymers. We calculated that PCL_75k_ contained 0.013 mmol/g of hydroxyl functional groups. Moreover, poly(αClεCL-*co*-εCL)_5k_ with 10 mol% of αClεCL units would contain 0.85 mmol/g of chloride functional groups, while sPCL_5k_ contained 0.80 mmol/g of hydroxyl groups. Addition of 75 wt.% poly(αClεCL-*co*-εCL)_5k_ to 25 wt% of PCL_75k_ increases the number of reactive groups in the melt by about 50 times (Table 2). Encouraged by these findings, we investigated the viability of the blending strategy for ME-AM applications and specifically whether the enhanced bulk functionality was reflected on the surface was investigated.

**Table 2:** Number of millimoles of reactive groups per gram in a melt of blends composed of different ratios of functionalized low and high molecular weight PCL polymers

Blend PCL_75k_: PCL_5k_	PCL_75k_ functional group: OH	poly(αClεCL-*co*-εCL)_5k_^b^ functional group: Cl	sPCL_5k_^a^ functional group: OH
(wt.%)	(mmol/g)	(mmol/g)	(mmol/g)
100:0	0.013		
75:25	0.010	0.21	0.20
50:50	0.007	0.43	0.40
25:75	0.003	0.64	0.60
0:100		0.85	0.80

^a^mmoles of hydroxyl groups in a blend based on sPCL_5k_ with four arms

^b^mmoles of chloride groups in a blend based on a 1:9 ratio of αClεCL to CL units in poly(αClεCL-*co*-εCL)_5k_. Average monomer molecular weight of the backbone unit: 117.4 mg/mmol

### Synthesis of polymers containing orthogonally reactive groups

As shown in Table 2, the theoretical increase in addressable functional groups (Cl and OH) via blending of either of the two functionalized low molecular weight polymers was similar. To obtain polymers with orthogonally reactive groups, the chlorides in poly(αClεCL-*co*-εCL)_5k_ were substituted for azide groups, and the hydroxyl end groups of sPCL_5k_ were converted via an esterification reaction using 6-maleimidohexanoic acid. Pursuing different chemical strategies permits exploration of their effects on the surface reactivity of functional groups. Although different polymer types were used (linear versus star polymers), we assumed that this would not significantly affect surface availability of the functional groups.

#### Synthesis of poly(αN_3_εCL-*co*-εCL)

**Scheme 1:**
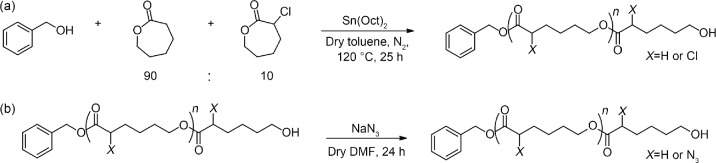
Poly(αN_3_εCL-*co*-εCL)_5k_ synthesis via **a** ring-opening polymerization of a 90:10 (mol:mol) εCL:αClεCL mixture and **b** subsequent substitution reaction with sodium azide. DMF: *N*,*N*-dimethylformamide

A linear PCL with azide groups on the backbone was synthesized in two steps. First, a ring-opening copolymerization reaction of εCL and αClεCL using stannous octoate as a catalyst was performed to yield poly(αClεCL-*co*-εCL). Subsequently, an S_N_2 reaction with sodium azide yielded poly(αN_3_εCL-*co*-εCL) (Scheme 1).

Following the pioneering work of the Jérôme group [42, 43], who studied the synthesis and ring-opening polymerization of different functionalized εCL monomers, a copolymerization of ε-CL and αClεCL was performed using tin(II) 2-ethylhexanoate (Sn(Oct)_2_) as a catalyst. The incorporation of αClεCL moieties is known to affect crystallinity, resulting in a lower *T*_m_ [42]. To ensure that the melting temperature remained above 37 °C, which is relevant for cell culture applications, we selected a copolymerization reaction using a monomer ratio of 90:10 (mol:mol). The ^1^H nuclear magnetic resonance (NMR) spectrum of the isolated copolymer showed signals of the caprolactoyl moieties at 4.06, 2.31, 1.65, and 1.38 ppm (Fig. S1 in Supplementary Information). Furthermore, at 4.26 ppm, a multiplet corresponding to the CH–Cl proton was present, and the presence of the CH–Cl stereocenter likely caused splitting of the signals of the adjacent methylene protons at 2.04 and 1.95 ppm. Integral ratios revealed that 11% of the polymeric units contained a chloro-substituent, which was similar to the feed molar ratio of 90:10 (entry 1 of Table S1 in Supplementary Information). Finally, evaluation of the thermal properties of the product revealed *T*_m_ of 44.3 °C, which is well above the temperature used during cell culture (Fig. S2a in Supplementary Information).

Next, we determined the molecular weight from the integral ratios of end groups and backbone protons afforded a *M*_n_ of 5.7 kg/mol, which was confirmed by gel permeation chromatography (GPC; entry 1 of Table S1 and Fig. S3 in Supplementary Information). Notably, the molecular weight of the polymer was lower than intended based on the monomer to initiator ratio. Despite rigorous drying of the solvent and monomers, we were unable to increase the molecular weight closer to the theoretical value (Sect. 2.1 and Figs. S4–S8 in Supplementary Information). The water present in the catalyst was likely difficult to remove, and no further experiments were performed to increase the polymer molecular weight, since approximately 5 kg/mol was suitable for this study.

Substitution of the chloride groups by azide functional groups was performed in DMF at RT (Scheme 1). Analysis of the ^1^H NMR spectrum revealed that the multiplet of the CH–Cl proton at 4.26 ppm disappeared and a new multiplet appeared at 3.83 ppm (Fig. S9 in Supplementary Information). Two-dimensional NMR analysis revealed the proximity of azide nitrogen atoms to this proton (Fig. S10 in Supplementary Information). The Fourier transform infrared spectroscopy (FT-IR) spectrum displayed an azide stretching vibration at 2100 cm^−1^ (Fig. S11 in Supplementary Information). GPC and differential scanning calorimetry (DSC) analysis showed that substitution yielded a product with a similar *M*_n_ and *T*_m_ (i.e., 46.3 °C) as that of poly(αClεCL-*co*-εCL) (Figs. S12a and S2 in Supplementary Information, respectively).

#### Synthesis of star-PCLM

**Scheme 2:**
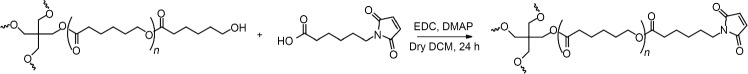
Star-PCL_5k_-maleimide (sPCLM) synthesis using EDC as a carboxylic acid activating agent and DMAP as a catalyst. EDC: 1-ethyl-3-(3-dimethylaminopropyl)-carbodiimide; DMAP: 4-dimethylaminopyridine; DCM: dichloromethane

End-functional maleimide groups were introduced onto the hydroxyl groups of sPCL_5k_ by reacting 6-maleimidohexanoic acid using 1-ethyl-3-(3-dimethylaminopropyl)-carbodiimide (EDC) as an activating agent and 4-dimethylaminopyridine (DMAP) as a catalyst (Scheme 2). ^1^H NMR evaluation revealed the almost full disappearance of methylene protons adjacent to the terminal hydroxyl group (3.65 ppm) as well as the appearance of maleimide protons at 6.65 ppm (Fig. S13 in Supplementary Information). We were able to obtain end-group conversion of up to about 90% by varying the stoichiometric excess of 6-maleimidohexanoic acid, EDC, and DMAP (Fig. S14 in Supplementary Information). GPC analysis showed a minor increase in the molecular weight of the isolated product, likely due to fractionation during precipitation (Fig. S12b in Supplementary Information). DSC measurements showed that *T*_m_ and crystallinity were both lower than that of the starting sPCL. Finally, thermal analysis revealed a decrease in *T*_m_ from 44.8 to 40.2 °C, and Δ*H* decreased from 51.6 to 33 J/g (Fig. S2b in Supplementary Information).

### Blends of PCL and poly(αClεCL-***co***-εCL) or sPCL

To prepare scaffolds with stable dimensions, a relatively high minimum viscosity of the melt is required for ME-AM applications [44]. If the viscosity is too low, the polymer will continue to flow after deposition until it has solidified into its intended shape. Changes in the viscosity and thermal properties in the polymer melt will affect printability, and thus we assessed the effect of blending on these properties. We first evaluated the change in viscosity in a qualitative screening experiment using a vial inversion test. Blends of sPCL_5k_ and PCL with intermediate molecular weight (PCL_31k_) at weight ratios of 25:75, 50:50, and 75:25 were prepared and heated to 60 °C. After 15 min after inversion, the 50:50 and 75:25 (wt.%) blends flowed, indicating a low melt viscosity. Similar results were obtained for the blends prepared from poly(αClεCL-*co*-εCL)_5k_ and PCL_31k_. Using a higher molecular weight PCL_75k_ yielded a much higher temperature threshold (i.e., 105 °C) for sufficient reduction of viscosity to enable flow of the melt (Fig. S15 in Supplementary Information). Thus, PCL_75k_ was used to prepare blends in further experiments.

Next, the thermal properties of all blends were investigated using DSC measurements. The *T*_m_ values of PCL_75k_, poly(αClεCL-*co*-εCL)_5k_, and sPCL_5k_ were 55.6, 44.3, and 44.8 °C, respectively (Table S2 and Fig. S2 in Supplementary Information). At a 75:25 blend ratio of PCL_75k_ to either poly(αClεCL-*co*-εCL)_5k_ or sPCL_5k_, a single melt transition was observed at about 55 °C. By contrast, at lower weight ratios of PCL_75k_, a bimodal melting peak was observed for all compositions (Fig. S16 in Supplementary Information). We suspected a potential cold crystallization process as result of the addition of poly(αClεCL-*co*-εCL)_5k_, which led to discrepancies between the crystallization enthalpy and the first melting enthalpy. For example, a PCL_75k_:poly(αClεCL-*co*-εCL)_5k_ at 25:75 (wt.%) showed a cooling transition at 22.6 °C with an enthalpy of 56 J/g, whereas the first melting peak (55.9 °C) had an enthalpy of 76 J/g (Table S2 in Supplementary Information). The occurrence of cold crystallization was confirmed by the disappearance of the bimodal peak of poly(αClεCL-*co*-εCL)_5k_, when the cooling rate was decreased to 1.5 °C/min, which provided sufficient time to re-form all crystalline domains (Fig. S2 in Supplementary Information). These results implied that more time is required for full crystallization when the amount of low molecular weight polymer is increased. This might affect the crystallinity of scaffolds, depending on their composition. Moreover, the gradual drift towards lower melting temperature indicated that different heating parameters might be required during extrusion.

**Fig. 2:**
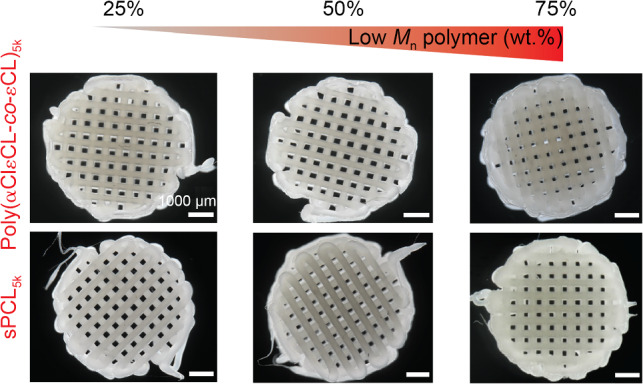
ME-AM scaffolds were prepared from different blended polymer ratios. Blends comprising up to 75 wt.% of poly(αClεCL-*co*-εCL)_5k_ (top) or sPCL_5k_ (bottom). Printing settings were optimized to obtain scaffolds with similar morphologies. Of note, extrusion of only low molecular weight polymers did not result in shape-stable scaffolds. ME-AM: melt extrusion-based additive manufacturing

Indeed, by pre-dominantly tweaking the extrusion temperature, scaffold manufacturing was successfully achieved from blends containing up to 75 wt.% of sPCL or poly(αClεCL-*co*-εCL) (Fig. 2). In addition, we confirmed that printing of the low molecular weight polymers alone did not afford shape-stable multilayered scaffolds. Accordingly, printer settings were optimized to obtain scaffolds with similar morphologies. The average fiber diameter and pore area of the 75:25, 50:50, and 25:75 PCL_75k_:poly(αClεCL-*co*-εCL)_5k_ scaffolds were (334±14), (309±19), (358±15) μm, and (569±28), (613±29), (526±40) μm^2^, respectively. In addition, the average fiber diameter and pore area of the 75:25, 50:50, and 25:75 PCL_75k_:sPCL_5k_ scaffolds were (300±17), (312±12), (315±12) μm, and (460±33), (540±53), (544±46) μm^2^, respectively (Fig. S17 in Supplementary Information).

**Fig. 3:**
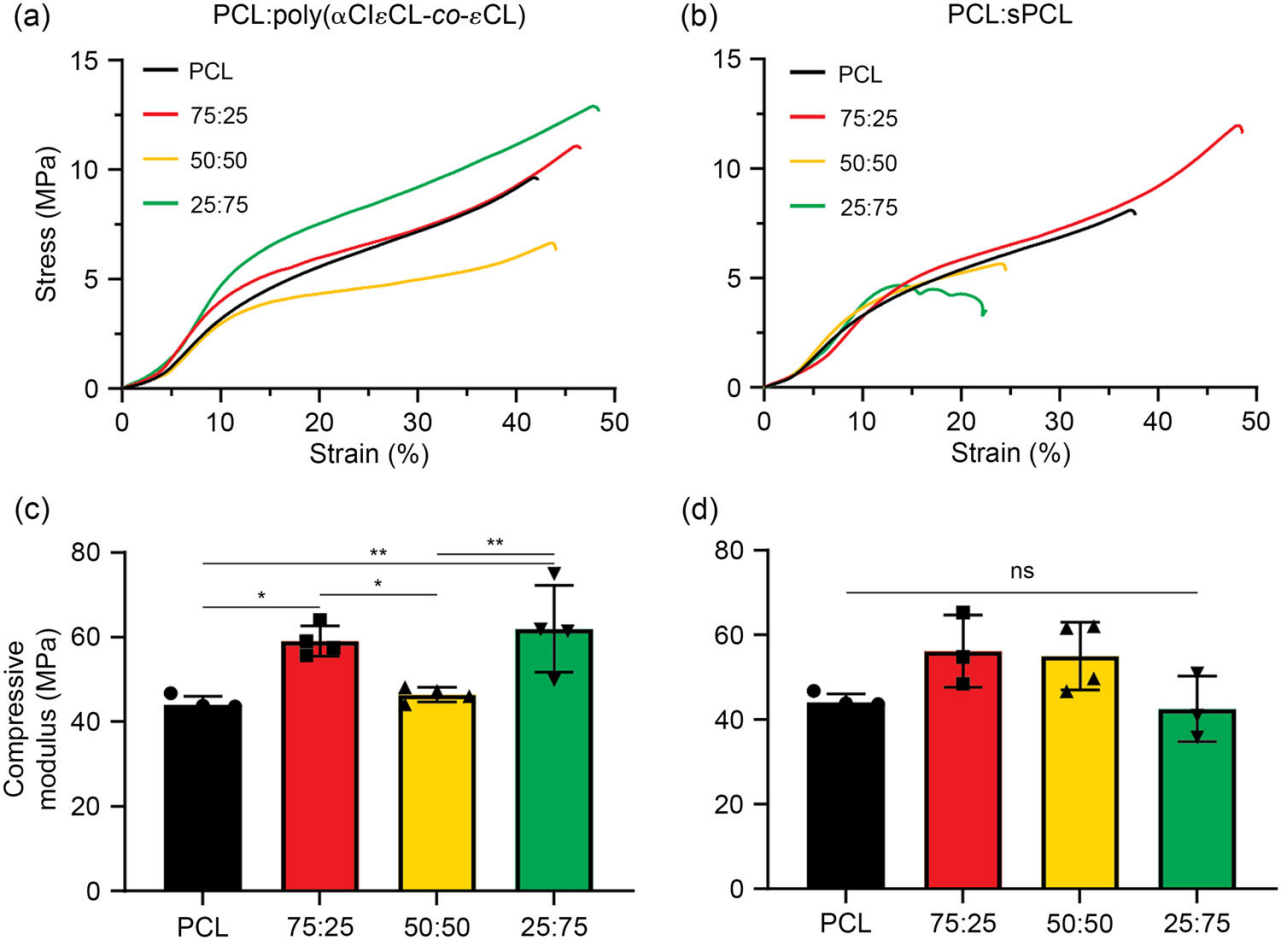
Mechanical properties of scaffolds in a compressive test. Representative stress–strain curves and compressive moduli of blends of poly(αClεCL-*co*-εCL)_5k_ and PCL_75k_ (**a**, **c**) and sPCL_5k_ and PCL_75k_ (**b**, **d**). All stress–strain curves can be found in Fig. S18 (Supplementary Information). Data are represented as mean±standard deviation (*n*≥3). ns: not significant; ^*^p<0.05; ^**^p<0.01

Subsequently, the mechanical properties of the ME-AM scaffolds prepared from all blend compositions were measured using compression tests. The stress–strain curves of these scaffolds resembled those of PCL_75k_ (Fig. 3 and Fig. S18 in Supplementary Information). All scaffolds, independent of composition, displayed a yield strain of approximately 14% and a yield stress of about 4 MPa (Fig. S19 in Supplementary Information). The moduli of scaffolds prepared from PCL_75k_ and poly(αClεCL-*co*-εCL)_5k_ ranged from (44±1) to (62±10) MPa (Figs. 3a and 3c). We observed slightly higher moduli of the 75:25 and 25:75 scaffolds, and this likely resulted from these scaffolds having a slightly larger fiber diameter (Fig. S17 in Supplementary Information). The compressive moduli of the scaffolds prepared from PCL_75k_ and sPCL_5k_ decreased from (56±9) to (43±8) MPa with decreasing PCL_75k_ content, although no statistically significant differences were observed (Figs. 3b and 3d). All blends showed a plateau after the elastic region followed by densification due to pore closure. The scaffold composed of PCL_75k_ and sPCL_5k_ at a weight ratio of 25:75 collapsed after yielding.

### Thermal stability and surface density of functional groups

In order to quantify the number of available functional groups on the surfaces, we opted to create thin films of each blend for characterization. Blends of PCL_75k_ and poly(αN_3_εCL-*co*-εCL)_5k_ or sPCLM_5k_ were prepared from CHCl_3_ solutions. After drying, films of the blends were prepared using a thermopress; this process simulates the heating and cooling process during ME-AM. Then, after functionalization, the films can be dissolved in a suitable solvent to quantify the number of functional molecules added to the surface (i.e., using a fluorescent dye). When testing thermopressing followed by dissolution, we noticed that the poly(αN_3_εCL-*co*-εCL)_5k_ blends were compatible with this approach, whereas the sPCLM_5k_ blends formed an insoluble core–shell like structure during dissolution. Possibly, the elevated temperature and pressure induced the formation of an insoluble network of cross-linked maleimides [45]. During ME-AM, blends would be subjected to high temperatures along with high pressure and shear forces. Therefore, the strategy that was relying on thiol-maleimide orthogonal chemistry for surface coupling was abandoned, and our focus shifted toward the blends containing poly(αN_3_εCL-*co*-εCL)_5k_. In future studies, the sPCL might be modified with a different type of reactive end group, such as an azide, that is compatible with the printing process.

**Fig. 4:**
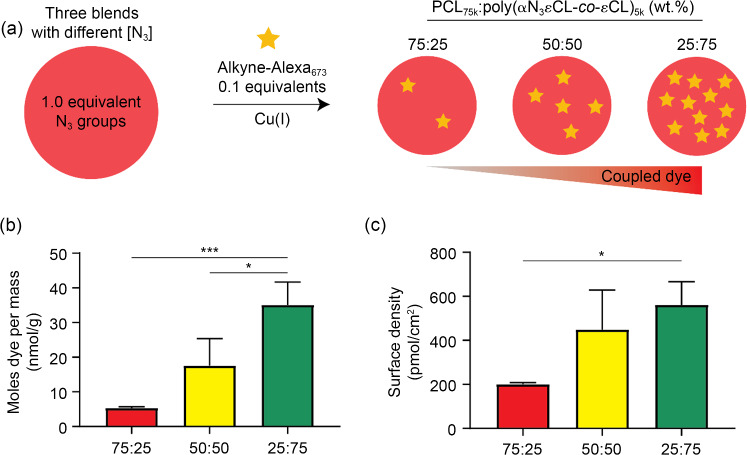
Determining the surface density of an alkynated model compound on films containing different surface azide densities. **a** An alkyne-Alexa_673_ dye was reacted on films prepared by blending PCL_75k_ and poly(αN_3_εCL-*co*-εCL) at weight ratios of 75:25, 50:50, and 25:75. The surfaces were dissolved in chloroform after washing and were then analyzed via spectrophotometry. All emission curves (*λ*_exc_=550 nm) are shown in Fig. S21 (Supplementary Information). From the emission curves, we determined the number of nanomoles of dye per gram of material **b** and surface density **c**. Data are represented as mean±standard deviation (*n*=3). ^*^p<0.05; ^***^p<0.001

Having confidence that the azide can withstand the printing process, we continued to prepare films of different ratios of PCL_75k_ and poly(αN_3_εCL-*co*-εCL)_5k_. These films were incubated in an aqueous solution containing a “clickable” dye (i.e., alkyne-Alexa_673_) to perform CuAAC chemistry on the surface (Fig. 4a and Scheme S1 in Supplementary Information). A control experiment was also performed to determine nonspecific binding; this test used 0.1 equivalents of alkyne-Alexa_673_ dye in the absence of the copper catalyst. Determined from a standard curve, we found that the concentration of the control sample was (15±6.0) nmol/L, which is about one fifth that of film containing the lowest amount of poly(αN_3_εCL-*co*-εCL)_5k_ (25%, (78±5.6) nmol/L) (Fig. S20a in Supplementary Information). Due to this apparent difference, we corrected the data for nonspecific absorption in further calculations.

The determined number of nanomoles per gram increased from (5.3±0.4) to (35±6.6) nmol/g, with increasing amount of poly(αN_3_εCL-*co*-εCL) present in the film (Fig. 4b). In addition, we found that increasing the amount of the dye from 0.1 to 0.25 equivalents of the azide groups did not lead to significant differences, suggesting that all groups present on the surface had reacted (Fig. S20b in Supplementary Information). Knowing the weight of the film and the surface area, the amount of the determined coupled dye was then used to calculate the surface density. Films containing 25%, 50%, or 75% of poly(αN_3_εCL-*co*-εCL) had surface densities of (201±7.7), (449±179), and (561±105) pmol/cm^2^ of coupled Alexa_673_ dye (Fig. 4c). In a previous study in which a bifunctional PCL containing azide groups was used, we reported a surface density of (32±6.3) pmol/cm^2^ [29]. This finding suggests that our work shows significant improvement. To place these values in perspective, we performed calculations to estimate the theoretical maximum density of surface-coupled peptides. We assumed that an average peptide covers an area of 0.25 nm^2^ (5 Å×5 Å, the area covered by about 10 amino acids). Accordingly, the maximum surface density of peptide molecules will then be 4×10^14^ per cm^2^. This estimated maximum corresponds to a surface density of approximately 670 pmol/cm^2^. Since the 25:75 film yielded a similar density (i.e., (561±105) pmol/cm^2^), we approached the theoretical maximum density, indicating the strength of the presented blending strategy to enhance both the bulk and surface functionality.

### Osteogenic differentiation of hMSCs

To assess the effect of peptide density on cell differentiation, a differentiation-inducing epitope of an alkynated BMP-2 peptide was reacted with films prepared from blends of PCL_75k_ and poly(αN_3_εCL-*co*-εCL)_5k_ at weight ratios of 75:25 and 25:75. These two weight ratios displayed the largest difference in surface density of the model dye (i.e., (201±7.7) and (561±105) pmol/cm^2^). The selected peptide is derived from the BMP-2 protein, which is known for its involvement in the osteogenic differentiation of seeded hMSCs [46, 47]. This BMP-2 protein has two receptor binding epitopes known as the “wrist” and “knuckle” sequences [48]. However, recent studies have questioned the intrinsic effect of the “wrist” epitope [29, 49], whereas enhanced osteogenesis has been achieved via the surface-grafted “knuckle” epitope [50–52]. Hence, the latter epitope was selected for this study. To begin the experiment, hMSCs were seeded for two weeks in basic medium (BM) or mineralization medium (MM) on films with and without the conjugated BMP-2 peptide.

**Fig. 5:**
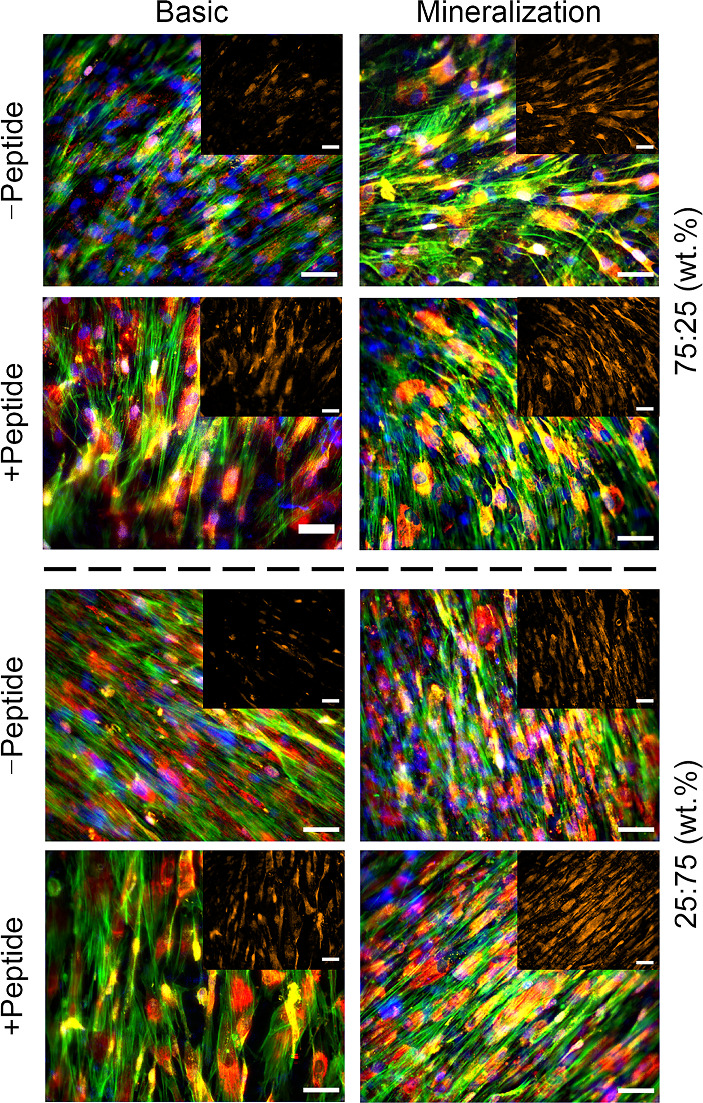
Fluorescent images of hMSCs seeded on top of 75:25 (top panel) and 25:75 (bottom panel) PCL_75k_:poly(αN_3_εCL-*co*-εCL) films with and without conjugated BMP-2-derived peptide. Images were taken after 14 days of culture in basic as well as mineralization media. The nuclei (blue), actin (green), BMPRII (yellow), and osteocalcin (red) were stained. The BMPRII channel is highlighted in the inset panel. Scale bar: 50 µm. hMSCs: human mesenchymal stromal cells; PCL: poly(ε-caprolactone); BMP-2: bone morphogenetic protein 2; BMPRII: BMP receptor II

Of the two epitopes, the knuckle shows greater affinity for BMPRII [53]. Binding to BMPRII leads to transphosphorylation of the BMP receptor I (BMPRI), inducing further downstream signaling pathways involved in osteogenic differentiation [54, 55]. Therefore, we performed an immunostaining after 14 days of culture to assess BMPRII expression (Fig. 5). In BM, the cells on surfaces without peptide displayed the lowest amount of BMPRII expression, whereas on surfaces with the conjugated peptide, the expression appears to be similar to cells cultured in MM. In MM, BMPRII was highly expressed independently on the presence of a surface-grafted peptide. Cells cultured in MM on pristine PCL films displayed an enhanced BMPRII expression compared to BM, further indicating that MM intrinsically activates BMPRII (Fig. S22 in Supplementary Information).

**Fig. 6:**
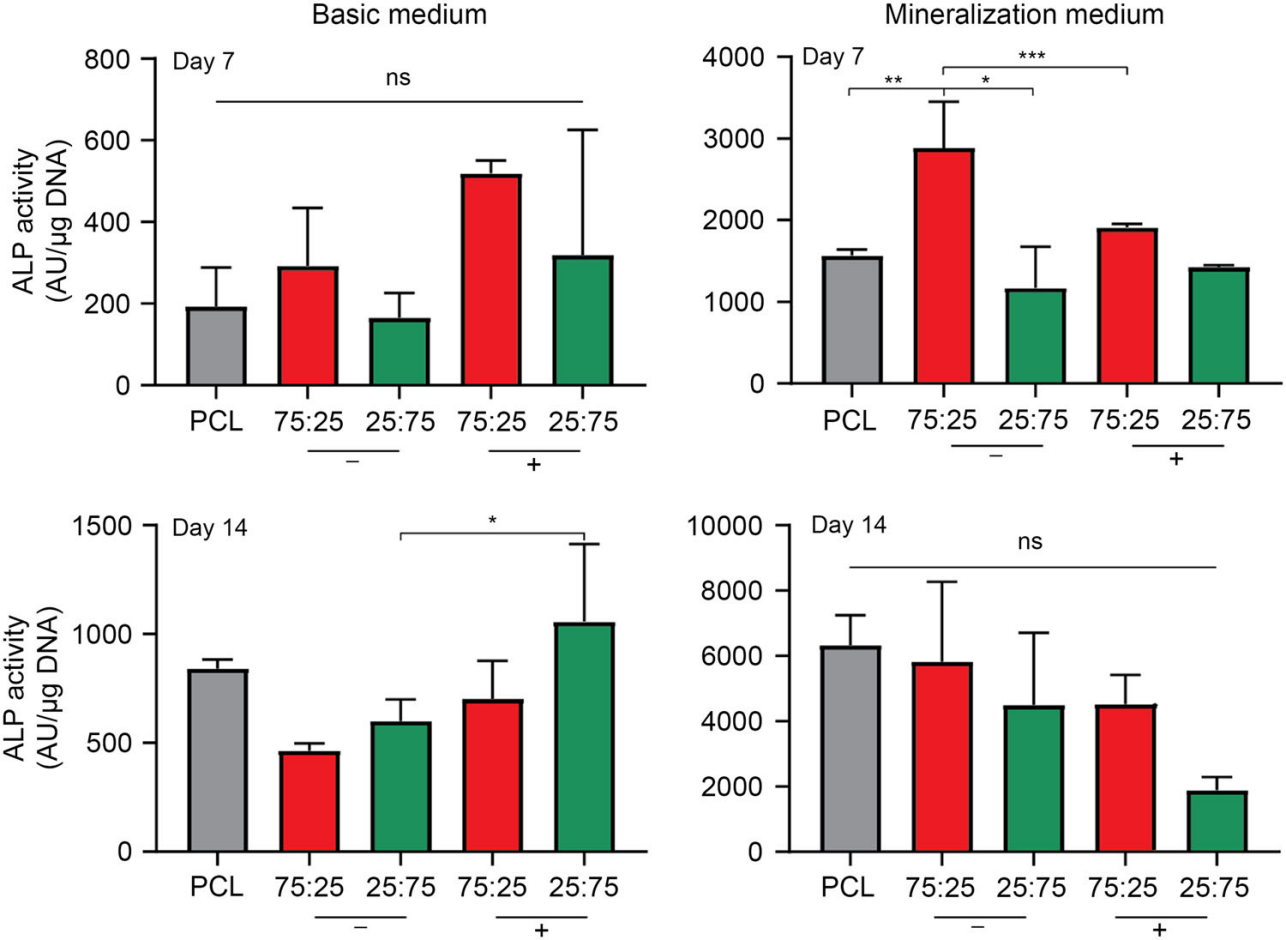
Normalized ALP expression of hMSCs after two weeks of differentiation in basic (left) and mineralization (right) media on PCL_75k_, and two blends (75:25 and 25:75) of PCL_75k_:poly(αN_3_εCL-*co*-εCL)_5k_ after 7 (top) or 14 (bottom) days. Cells were cultured on surfaces without (−) or with (+) alkynated BMP-2 peptide. Data are represented as mean±standard deviation (*n*≥3). ns: not significant; ^*^p<0.05; ^**^p<0.01; ^***^p<0.001. ALP: alkaline phosphatase; hMSCs: human mesenchymal stromal cells; PCL: poly(ε-caprolactone); BMP-2: bone morphogenetic protein 2

ALP is an early osteogenesis marker associated with increased matrix mineralization [56]. After seven days in BM, we observed higher normalized ALP expression on films with grafted peptides. After 14 days in BM, conjugation of the peptide on the 75:25 films led to an increased normalized ALP expression from (4.6±0.3)×10^2^ (no peptide) to (7.0±1.7)×10^2^ AU/µg DNA (with peptide). On the 25:75 films, we also observed a significant increase in normalized ALP expression on surfaces with conjugated peptides, from (6.0±1.0)×10^2^ (no peptide) to (10.0±3.5)×10^2^ AU/µg DNA (with peptide). In MM, all values plateaued after 14 days at approximately 5.0×10^3^ AU/µg DNA. Notably, on 25:75 films with conjugated peptide in MM, a decline was observed at this time point; however, statistical analysis revealed that this difference was not statistically significant (Fig. 6). Overall, the trends in ALP activity align well with the changes in BMPRII expression.

Next, we assessed the expression of osteocalcin, a late-stage marker of osteogenic differentiation [57, 58]. In the immunofluorescent images, osteocalcin was present on all surfaces after 14 days (Fig. 5). Notably, we found that hMSCs cultured on tissue culture plastic (TCP) in BM also displayed osteocalcin (Fig. S23 in Supplementary Information). These results may be related to the high cell density on the films and TCP, as shown by the F-actin staining.

The concentration-dependent effect of the “knuckle” epitope of the BMP-2 peptide on osteogenic differentiation of hMSCs has been described by previous studies. For example, the Becker group used a strategy to create a BMP-2 peptide gradient on glass coverslips based on orthogonal click chemistry; this study covered a surface density of 40–130 pmol/cm^2^ [59, 60]. Using a strategy based on the same principles as those of the Becker group, Bilem and coworkers obtained a BMP-2 surface density in the range of 40–220 pmol/cm^2^ [61, 62]. In both studies, osteogenic differentiation markers such as RUNX2 (early) and bone sialoprotein (late) were enhanced only in the 100–220 pmol/cm^2^ range, clearly illustrating the effect of surface density. In this study, we covered a surface density range of 201–561 pmol/cm^2^. We also observed that from 200 pmol/cm^2^ onward, BMPRII expression was enhanced, indicating that osteogenic differentiation was activated because of the peptide. Notably, ALP expression was only significantly upregulated on the 25:75 films (561 pmol/cm^2^). Since previous studies used a glass substrate instead of a polymeric surface, the optimal concentration of peptide on the surface might differ depending on the material of the substrate.

## Conclusions and future directions

In this study, we present a general strategy for tuning the density of reactive groups on the surface of ME-AM scaffolds. This strategy was based on the blending of low molecular weight PCL-derived polymers (i.e., poly(αClεCL-*co*-εCL)_5k_ and sPCL_5k_) and high molecular weight PCL_75k_. Because blending of up to 75% (mass fraction) low molecular weight polymer afforded stable scaffolds, we conclude that the strategy is suitable for biofabrication applications using ME-AM. In contrast to azidated polymers, the use of polymers with maleimide end groups was not possible due to their thermal instability. Therefore, we used varying ratios of poly(αClεCL-*co*-εCL)_5k_ in blends to prepare thermopressed films to illustrate that the surface density can be tuned. Using three polymer ratios, we covered a range of surface densities from 201 to 561 pmol/cm^2^, which implies that further optimization can unlock an even broader range.

Finally, we investigated the effect of surface density of BMP-2-derived peptides on osteogenic differentiation of hMSCs seeded on films. We found that both BMPRII and ALP activity had a higher expression in BM on the BMP-2 peptide functionalized films. Notably, ALP activity was only significantly upregulated on the surface comprising the highest peptide density. After 14 days of culturing, we also observed the expression of osteocalcin. Based on these results and those of previous reports, the threshold surface density of the “knuckle” BMP-2-derived epitope to induce osteogenesis appears to be at least 100 pmol/cm^2^. Thus, these results highlight the fact that the surface density of grafted peptide molecules is related to cell fate. Future studies that functionalize surfaces of AM scaffolds should take the surface density into account as a higher degree of control over cell differentiation may be gained; such an advance may push tissue engineered scaffolds closer towards the clinical.

Taken together, we consider the reported strategy as a proof of concept and envision that other polymers as well as hydrogel systems can use blending to enhance the density of reactive groups. Moreover, due to the versatility of “click” chemistry, the presented strategy permits the incorporation of a wide variety of biomolecules, making these findings of interest for the broader tissue engineering community. In addition, bio-orthogonal chemistry presents an opportunity for use in the surface functionalization of ME-AM scaffolds comprising compositional gradients, which is important for the regeneration of tissue interfaces.

### Supplementary Information

Below is the link to the electronic supplementary material.

Supplementary file1 (PDF 2255 kb)

## Data Availability

The data that support the findings of this study are openly available in DataverseNL at Ref. [63].
