# Supplementary material for: A facile strategy for tuning the density of surface-grafted biomolecules for melt extrusion-based additive manufacturing applications
Source: Biodes Manuf. 2024 May 20;7(3):277–91. doi: 10.1007/s42242-024-00286-2 (PMC11133161; doi:10.1007/s42242-024-00286-2)
Supplement: Supplementary file 1 — Supplementary file1 (PDF 2255 kb) [file 42242_2024_286_MOESM1_ESM.pdf]

Supporting Information

A facile strategy for tuning the density of surface-grafted biomolecules for melt extrusion based additive manufacturing applications

Authors

I.A.O Beeren[1], G. Dos Santos[1,2,3], P.J. Dijkstra[1], C. Mota[1], J. Bauer[1], H. Ferreira[2,3], R. Reis[2,3], N. Neves[2,3], S. Camarero-Espinosa[1,4,5], M.B. Baker[1], L. Moroni[1*]

[*]Corresponding author email address: l.moroni@maastrichtuniversity.nl

Affiliations

1 Department of Complex Tissue Regeneration, MERLN Institute for Technology-Inspired Regenerative Medicine, Maastricht University, 6229 ER Maastricht, The Netherlands

2 3B's Research Group, I3Bs – Research Institute on Biomaterials, Biodegradables and Biomimetics, University of Minho, Headquarters of the European Institute of Excellence on Tissue Engineering and Regenerative Medicine, AvePark, Zona Industrial da Gandra, 4805-017 Barco, Guimarães, Portugal

3 ICVS/3B's-PT Government Associate Laboratory, 4805-017 Braga/Guimarães, Portugal

4 POLYMAT, University of the Basque Country UPV/EHU, 20018 Donostia/San Sebastián, Spain

5 IKERBASQUE, Basque Foundation for Science, 48009 Bilbao, Spain

Table of contents

1. Supplementary Methods 3

1.1 Synthesis 3

1.2 Methods 4

2. Chemical characterization of polymers 5

2.1 Further results and discussion on the poly(αClεCL-*co*-εCL) synthesis 8

2.2 Poly(αN_3εCL-*co*-εCL) characterization 14

2.3 sPCLM characterization 17

3. Scaffold characterization 18

4. Characterization of surface-grafted dyes 22

5. hMSC differentiation on thermopressed films containing Poly(αN_3εCL-*co*-εCL)$_{5k}$ with surface-grafted BMP2 peptide 24

6. References 24

1. Supplementary Methods

1.1 Synthesis

Poly(αClεCL):

In an oven-dried flask and under a dry N_2 atmosphere, 500 mg αClεCL (3.36 mmol, 350 equiv.) and 1.0 µL benzylic alcohol ($9.6 \cdot 10^{-3}$ mmol, 1.0 equiv.) were added. Subsequently, 1 mL of anhydrous toluene and 0.5 mg $Sn(Oct)_2$ ($1.2 \cdot 10^{-3}$ mmol, 0.125 equiv., 2.85 $mg.mL^{-1}$ stock solution in dry toluene) were added. The resulting mixture was purged for 30 min with dry N_2 and left for 24 h at 120 °C. The mixture was diluted with DCM and the polymer was precipitated in cold methanol. The product was collected as a viscous transparent liquid. Yield; 0.16 g, 32%.^{1}H NMR (700 MHz, $CDCl_3$): δ 1.51 (m, 92H), 1.59 (m, 107H), 1.73 (m, 159H), 1.97 (m, 80H), 2.05 (m, 80H), 3.67 (t, 2H, J = 6.3 Hz), 4.20 (m, 157H), 4.27 (m, 76H), 5.21 (s, 0.5H), 5.30 (DCM, s, 0.2H), 7.3 (m, 1.1H). GPC: 12.0 $kg \cdot mol^{-1}$.

Star-PCLM:

In a typical reaction, $sPCL_{5k}$ (3.3 g, 2.6 mmol of hydroxyl groups, 1.0 equivalents) was dried on a Schlenk line in an oven-dried round flask containing 6-maleimidohexanoic acid (4.5 g, 21.1 mmol, 8.0 equivalents), 1-ethyl-3-(3-dimethylaminopropyl)carbodiimide hydrochloride salt (EDC, 2.0 g, 10.6 mmol, 4.0 equivalents), and 4-dimethylaminopyridine (DMAP, 0.13 g, 1.06 mmol, 0.4 equivalents) overnight. Subsequently, 66 mL of anhydrous DCM was added, and the reaction mixture was stirred at RT for 24 h under a N_2 atmosphere. To vary the degree of substitution with maleimide, we also reacted sPCL-OH with different stoichiometric amounts of 6 maleimidohexanoic acid, EDC, and DMAP, namely 4:2:0.2 and 2:1:0.1 equivalents.

After evaporation of the solvent under reduced pressure, the concentrated mixture was precipitated in methanol. The obtained polymer was dissolved in $CHCl_3$ and precipitated in methanol and this process was repeated at least three times. The product was collected as a pale red solid. Yield; 3.0 g, 90%. ^{1}H NMR (700 MHz, $CDCl_3$): δ 1.38 (q, 20H, J = 7.6 Hz), 1.64 (m, 46H), 2.31 (t, 24H, J = 7.6 Hz), 3.51 (t, 1.8H, J = 7.1 Hz), 3.66 (b, 0.8H), 4.06 (t, 20H, J = 6.7 Hz), 4.11 (s, 2H), 6.69 (s, 1.3H). GPC: 13.5 $kg \cdot mol^{-1}$, Đ = 1.30. DSC: T_m = 40.3 °C.

1.2 Methods

Nuclear magnetic resonance (NMR) spectroscopy:

All samples were dissolved in deuterated chloroform. ^{1}H NMR and heteronuclear multiple bond correlation (HMBC) spectra were recorded at 299.7 K using a Bruker Ascend 700 MHz NMR spectrometer and analyzed with TopSpin 4.0 software (Bruker, Germany).

Gel permeation chromatography (GPC):

Molecular weights and dispersities were determined by GPC using a Prominence-I LC-2050C3D LC (Shimadzu) system comprising of an autosampler, a Shim-pack GPC 800P guard (4.6 x 10 mm) column, followed by a Shim-pack 80M (8.0 x 300 mm) column, a refractive index detector, and a photodiode array detector. Tetrahydrofuran (THF) was used as mobile phase at a flow rate of 1.0 ml.min^{-1} at 40 °C. Polystyrene standards were used for calibration. Samples were dissolved at 1 mg.mL^{-1} in THF, filtered, and 50 µL was injected for analysis.

Attenuated Total Reflectance Fourier Transform Infrared (ATR-FTIR) spectroscopy:

ATR-FTIR spectra were recorded on a Nicolet iS50 FT-IR instrument (ThermoFisher) in the range of 4000–400 cm^{-1}. After recording 16 scans for background correction, samples were measured applying 32 scans.

Differential scanning calorimetry (DSC):

Thermal properties of homopolymers, copolymers, and blends were determined using a TA instrument DSC250. Samples (~5.0 mg) were placed in aluminum pans and, under a N_2 flow, heated from 30 ºC to 100 ºC at a rate of 10 ºC.min^{-1}. The samples were held isothermal for 5 min to erase thermal history. $sPCL_{5k}$, $sPCLM_{5k}$, poly(αClεCL-*co*-εCL)$_{5k}$, poly(αN_3εCL-*co*-εCL)$_{5k}$ were cooled to -80 ºC at -1.5 ºC.min^{-1}. All used blend ratios were subjected to a cooling rate of -5 ºC.min^{-1}. After keeping the samples isothermal for 1 min, they were heated again to 100 ºC at 5 ºC.min^{-1}. The thermal properties were determined from the cooling and second heating cycle.

2. Chemical characterization of polymers

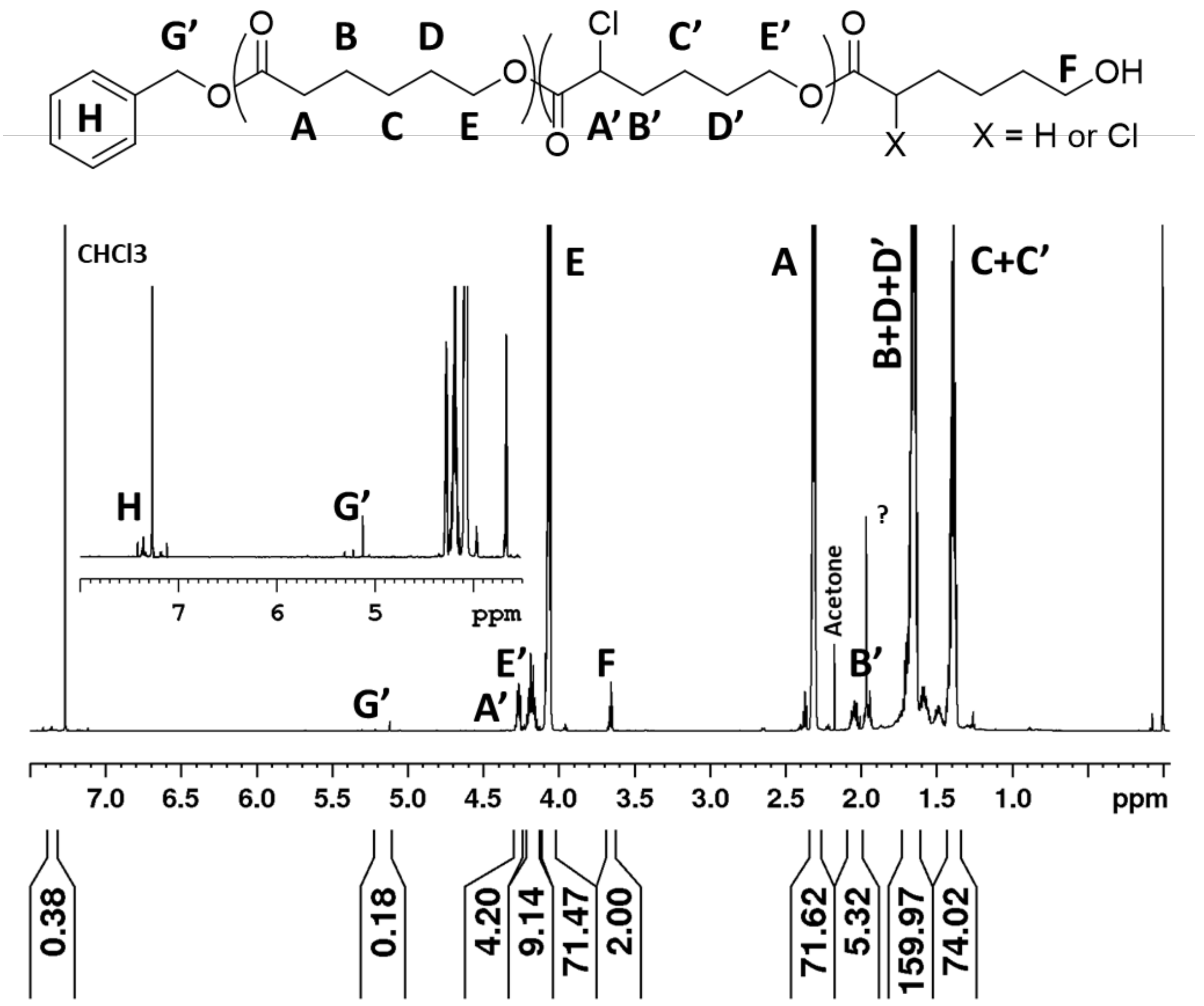

Figure S1. [1]H Nuclear Magnetic resonance (NMR) spectrum (700 MHz, $CDCl_3$) of poly(αClεCL-*co*-εCL) synthesized according to the conditions specified in entry 1 (See Table S1).

Table S1. Synthesis poly(αClεCL-*co*-εCL) via a ring-opening polymerization of αClεCL and εCL using benzylic alcohol as initiator and $Sn(Oct)_2$ as catalyst. εCL was dried over CaH_2, and stored over molecular sieves (3Å). Toluene was dried for 24 h over molecular sieves (3Å).

Entry[a,b]	Degassing method	Drying αClεCL	$F_{\alpha Cl\varepsilon CL}$ (%)[c]	$M_{n,\ NMR}$ (kg.mol^{-1})[d]	$M_{n,\ GPC}$ (kg.mol^{-1})[e]
1	Freeze thaw	None	11.4	5.7	6.7
2	x	Azeotropic distillation	35	12	15.0
3	x	Sieves 3Å	8.2	7.9	14.3
4	x	Silica plug	13.7	5.6	14.5

a) The stoichiometry of $Sn(Oct)_2$: benzylic alcohol : αClεCL : εCL was kept constant at 0.125 : 1.0 : 50 : 450 equivalents during the reaction. The monomer concentrations were kept constant at 0.4 M and 3.4 M, respectively.

b) The theoretical maximum molecular weight of polymer was calculated based on the molar feed ratio and the molecular weight of the repeating unit (αClεCL or εCL), according to $M_{n, theoretical}$ = 50*148,9 + 450*114,14 = 58.8 kg.mol^{-1}.
c) The degree of chloride incorporation in the backbone was determined via ^{1}H NMR. The protons adjacent to the hydroxyl groups (3.65 ppm) were set to 2.0. Then, the integral of proton adjacent to chloride (4.26 ppm) was divided by the integral of the backbone (1.38 ppm), according to $I_{A'}/(I_C/2)$*100.
d) The M_n was determined via ^{1}H NMR (700 MHz, $CDCl_3$). The integral of the methylene protons adjacent to the hydroxyl end group was set to 2.00. Then, the unit molecular weight of the εCL and αClεCL were multiplied by the normalized integral value of the backbone, according to $(I_E/2)$*114.14 + $(I_{A'}/1)$*148.9. The ^{1}H NMR spectra of all entries can be found in figure S5–S7.
e) The M_n of purified products was determined by Gel Permeation Chromatography (GPC) using THF as eluent. Retention curves can be found in figure S3.

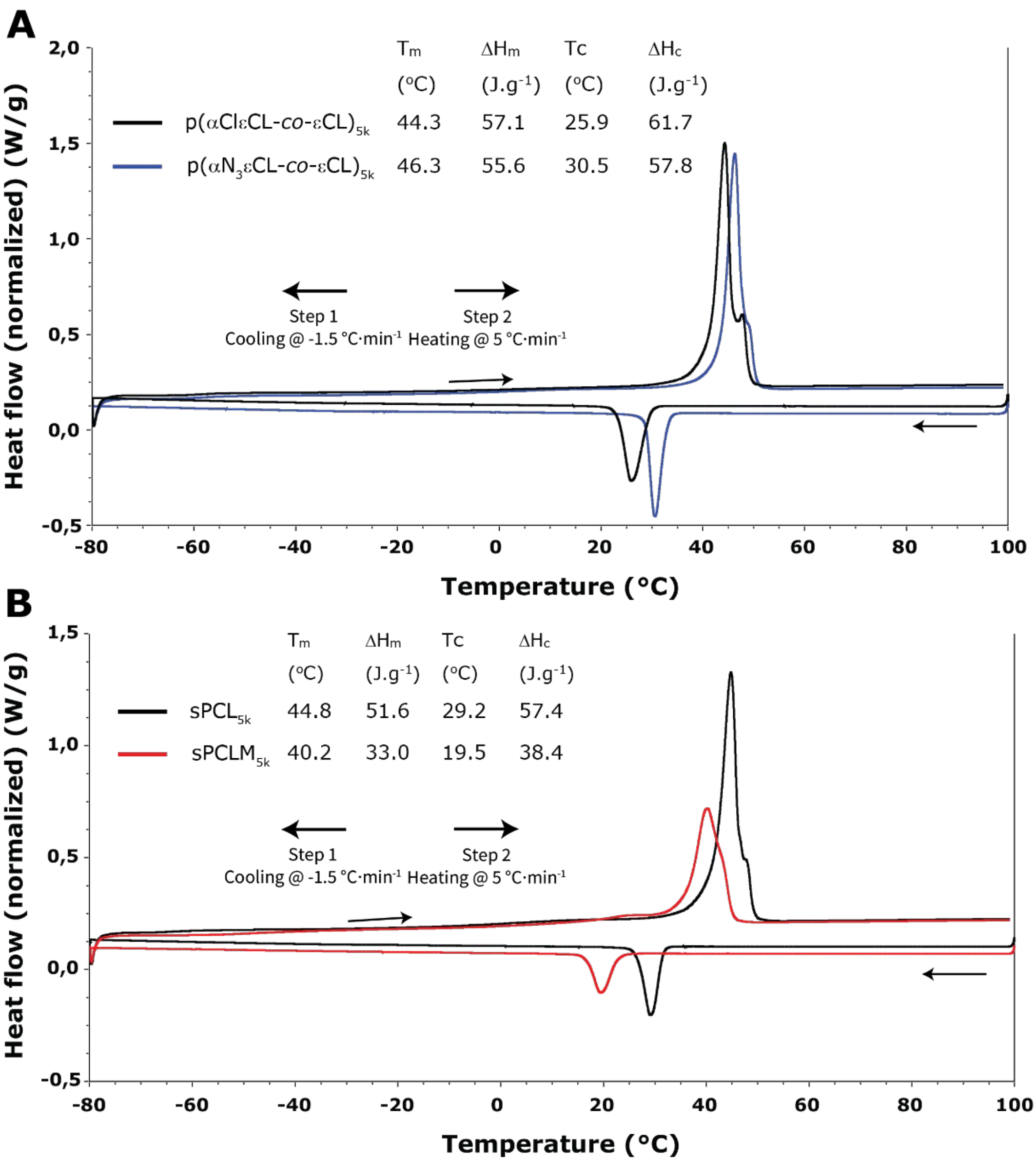

Figure S2. DSC heating and cooling thermograms of (A) p(αClεCL-*co*-εCL) and p(αN_3εCL-*co*-εCL) and (B) sPCL and sPCLM. Samples were heated to 100 °C, and kept isothermal for 5.0 min to remove thermal history. Then, as depicted in the graphs, the samples were cooled at -1.5 °C.min^{-1} and heated at 5 °C.min^{-1}. The thermal properties were determined from the cooling and second heating cycle.

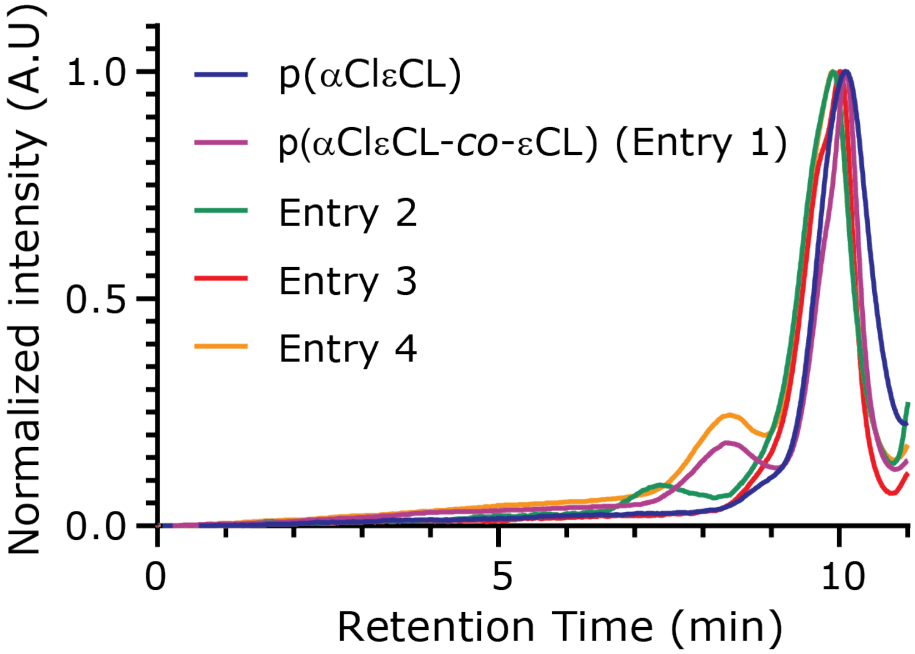

Figure S3. GPC traces using THF as eluent of the homopolymerization of αClεCL and p(αClεCL-*co*-εCL) of entry 1–4 (Table S1).

2.1 Further results and discussion on the poly(αClεCL-*co*-εCL) synthesis

To synthesize the poly(αClεCL-*co*-εCL), we performed a ring opening copolymerization reaction of ε-CL and αClεCL in a 90 to 10 mol% feed ratio using benzylic alcohol as initiator. We initially set out to yield a high molecular weight polymer with an estimated molecular weight of 58.8 $kg.mol^{-1}$. However, when we used the methylene protons adjacent to hydroxyl end groups, the molecular weight was drastically lower (Table S1). Simultaneously, we did observe full monomer conversion in 25 h. These results implied the presence of water in the reaction mixture.

In earlier work, we polymerized only εCL using $Sn(Oct)_2$ as catalyst, and the expected molecular weight was approached [1]. Therefore, we investigated if the αClεCL was the source of water. We performed a homopolymerization of αClεCL using the same reaction conditions as the copolymerization. According to structural analysis of NMR, we successfully obtained the homopolymer poly(αClεCL). Based on integration ratio of end-group versus backbone, we found a molecular weight of ~12 $kg.mol^{-1}$ (Figure S8), which was roughly 4–5 times lower than expected. Next, we attempted more rigorous drying steps of the αClεCL (Table S1). All methods did not significantly change the molecular weight (Figure S3 & Table S1). Due to process of elimination, we concluded that the catalyst was the most likely source of the water.

Nevertheless, we obtained the desired molecular weight of ~5 $kg.mol^{-1}$ for this study. To demonstrate control over the reaction with the current set of chemicals, we added half the amount of initiator (0.5 equiv.), and observed an approximate doubling of the molecular weight, from 6.7 to 11.3 $kg.mol^{-1}$ (Figure S10). In future studies, we recommend to consider switching to Zinc-based catalysts such as zinc bisamide as they are generally easier to dry. This type of catalyst was already used in a successful copolymerization of αClεCL and εCL [2].

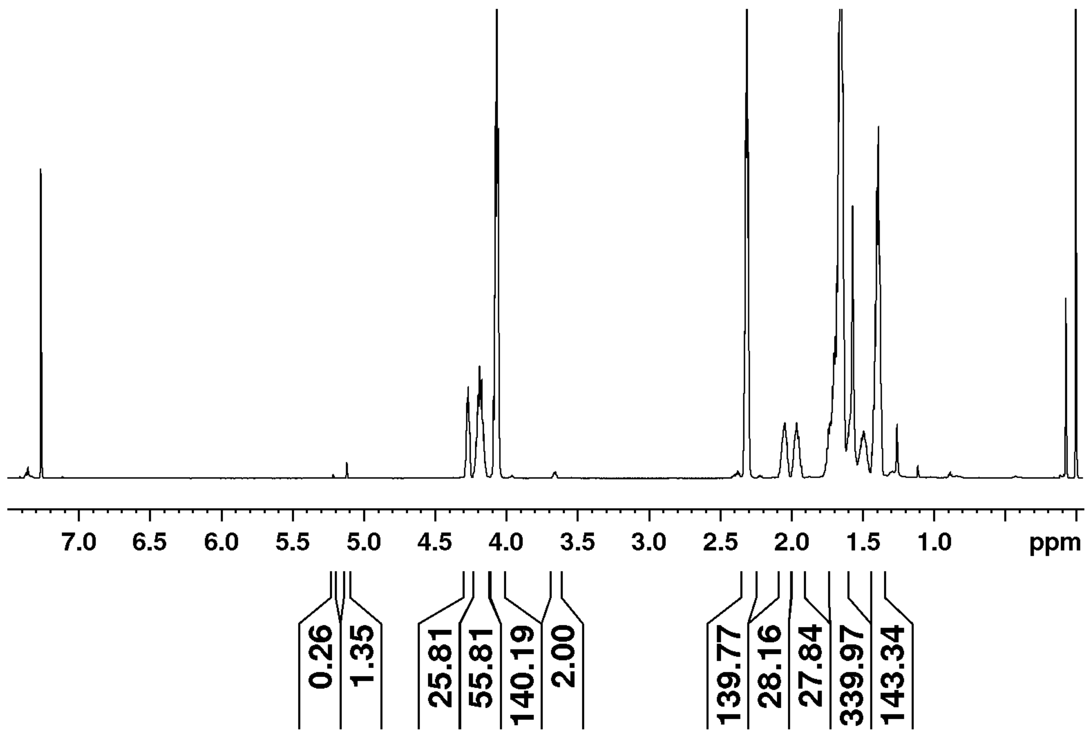

Figure S4. ^{1}H NMR spectrum (700 MHz, $CDCl_3$) of purified poly(αClεCL-*co*-εCL) according to entry 2 (See Table S1).

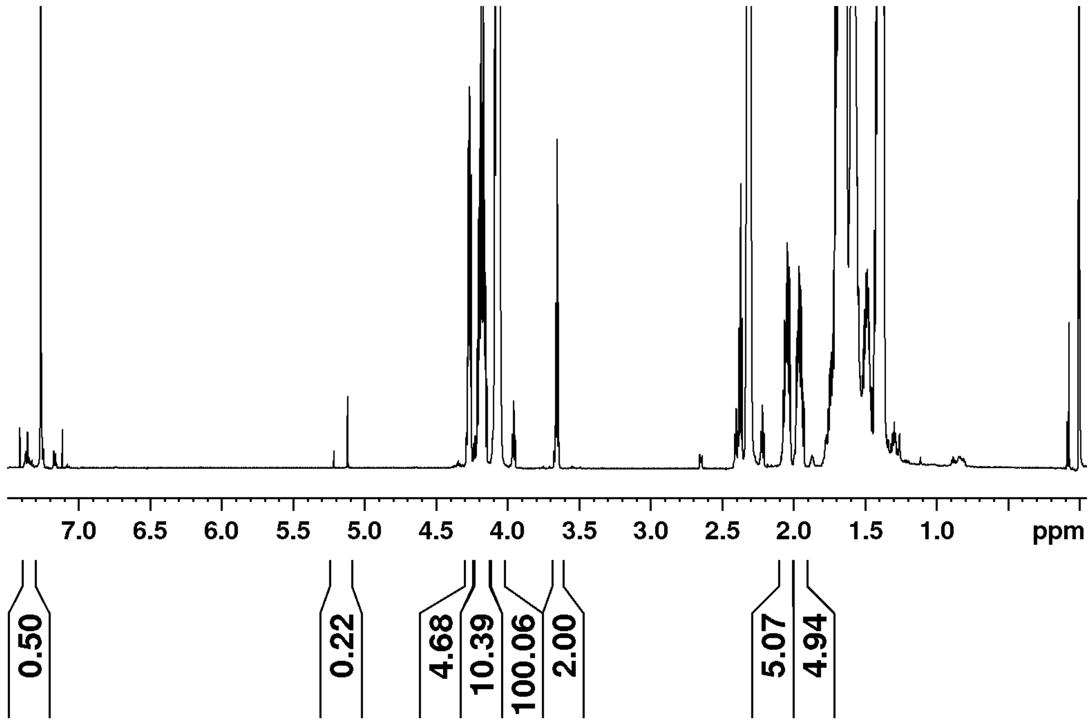

Figure S5. ^1H NMR spectrum (700 MHz, $CDCl_3$) of purified poly(αClεCL-*co*-εCL) according to entry 3 (Table S1).

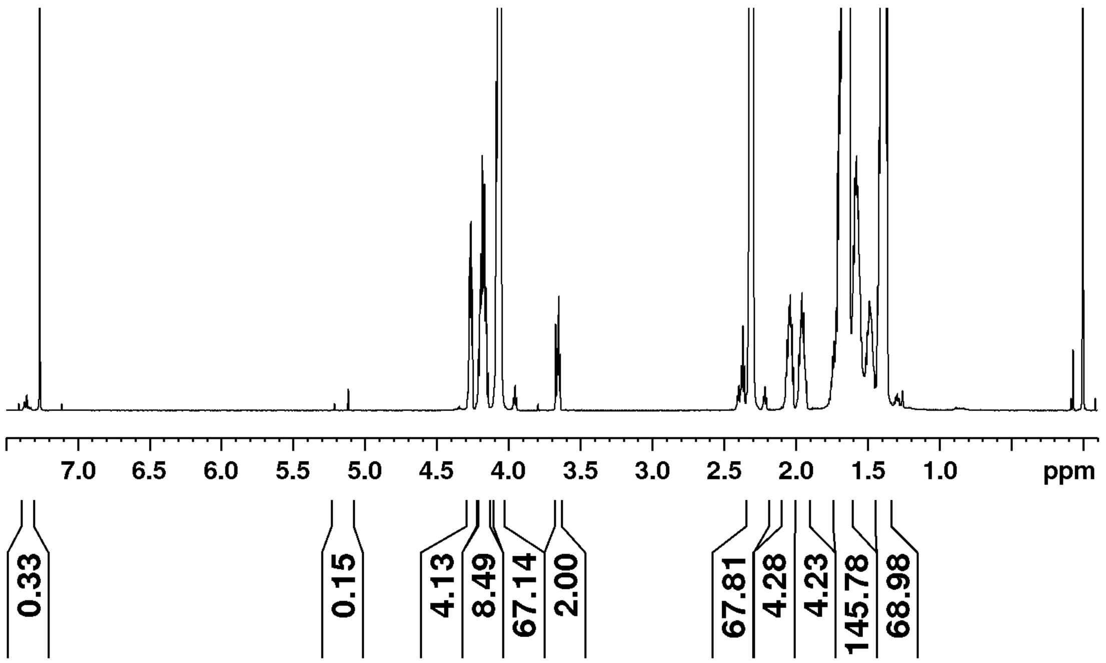

Figure S6. ^{1}H NMR spectrum (700 MHz, $CDCl_3$) of purified poly(αClεCL-*co*-εCL) according to entry 4 (Table S1).

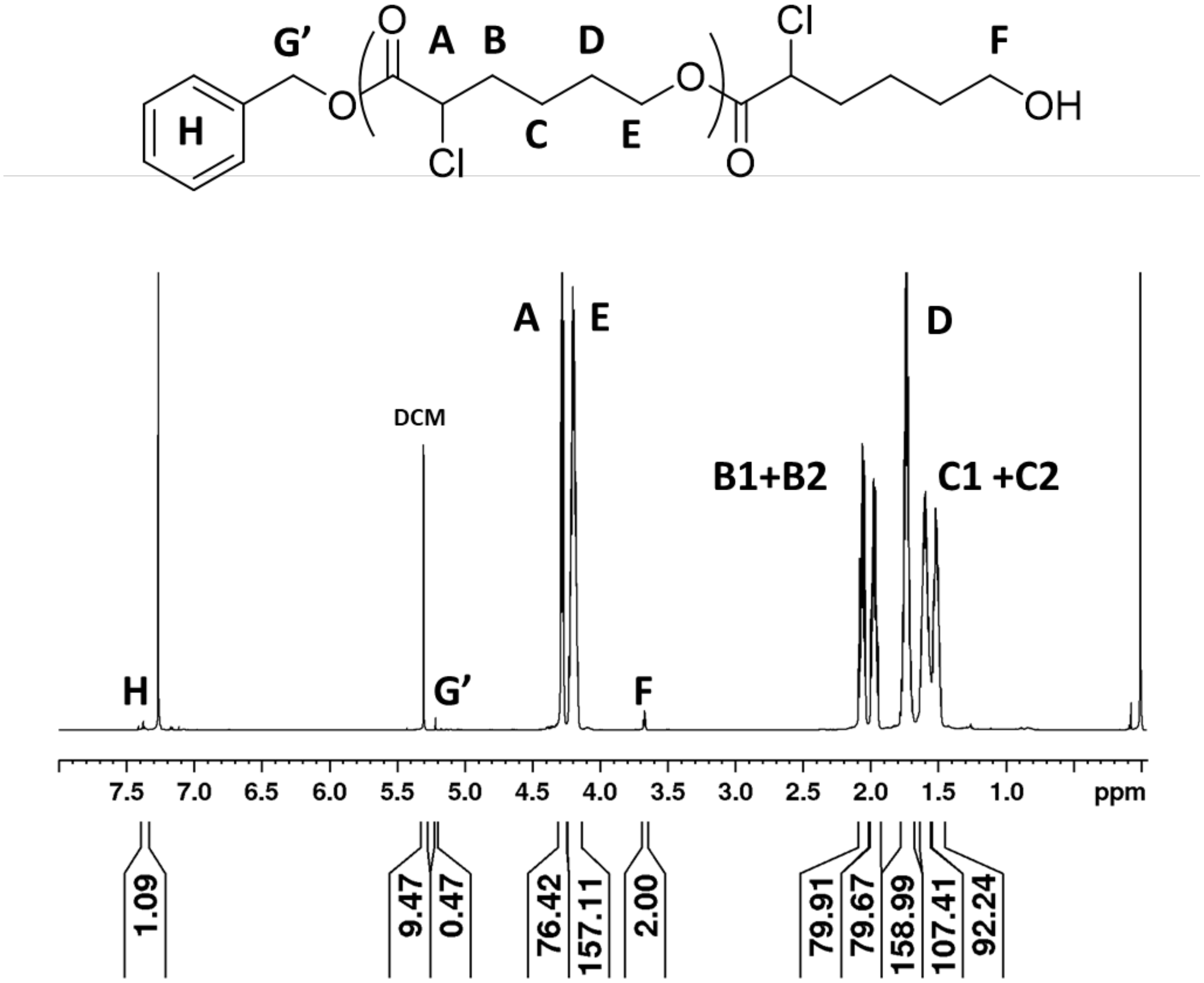

Figure S7. ^{1}H NMR spectrum (700 MHz, $CDCl_3$) of homopolymer poly(αClεCL).

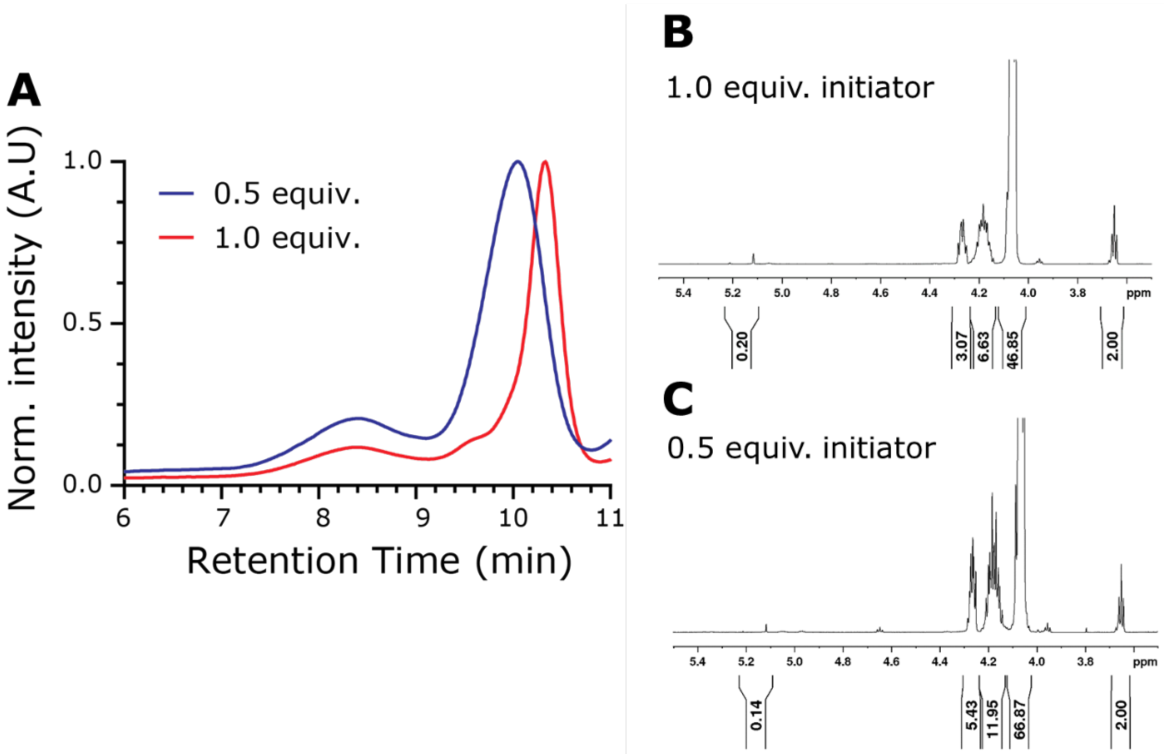

Figure S8. A) GPC traces of poly(αClεCL-*co*-εCL) synthesized according to entry 1 of Table S1 (red), and entry 1 using half (0.5 equiv.) the amount of initiator (blue). (B) ^{1}H NMR spectrum (700 MHz, $CDCl_3$) of poly(αClεCL-*co*-εCL) synthesized using either (B) 1.0 or (C) 0.5 equivalents of initiator. Both GPC and NMR indicated roughly a doubling of the molecular weight when the amount of initiator was halved.

2.2 Poly(αN$_3$εCL-*co*-εCL) characterization

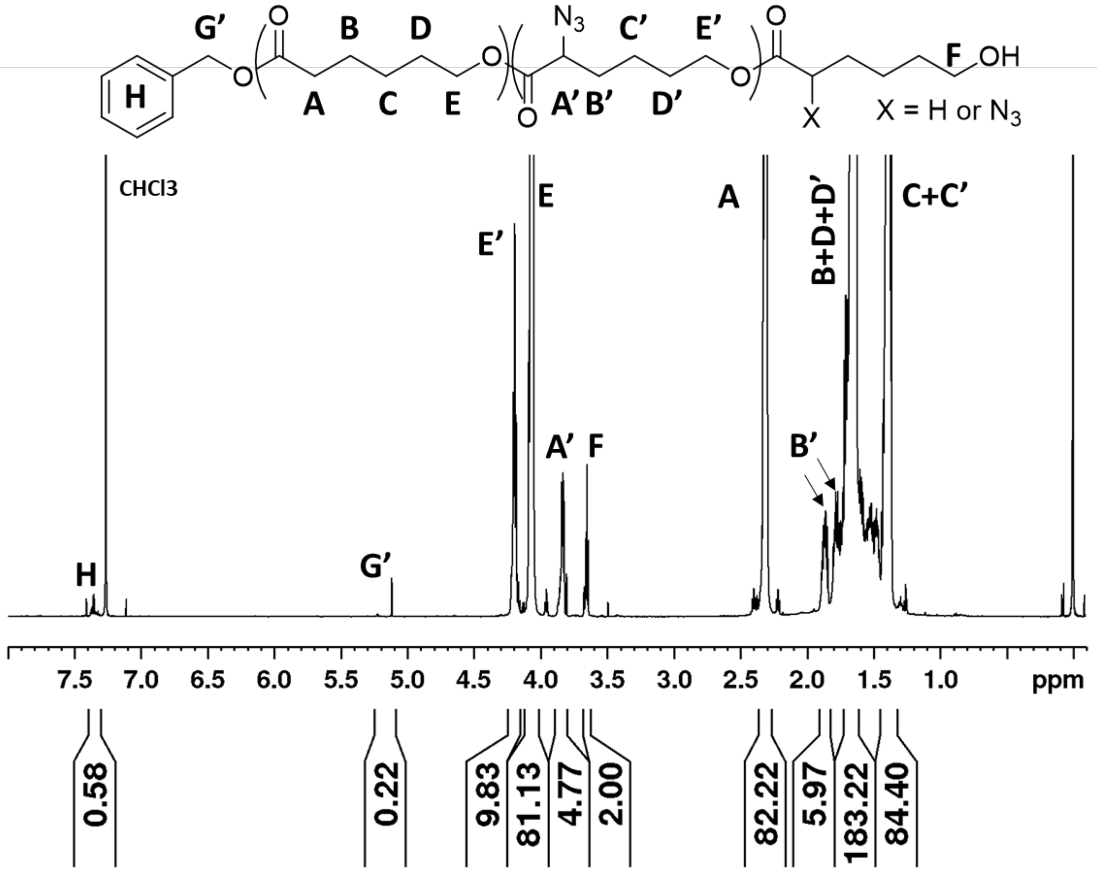

Figure S9. ^{1}H NMR spectrum (700 MHz, $CDCl_3$) of poly(αN$_3$εCL-*co*-εCL).

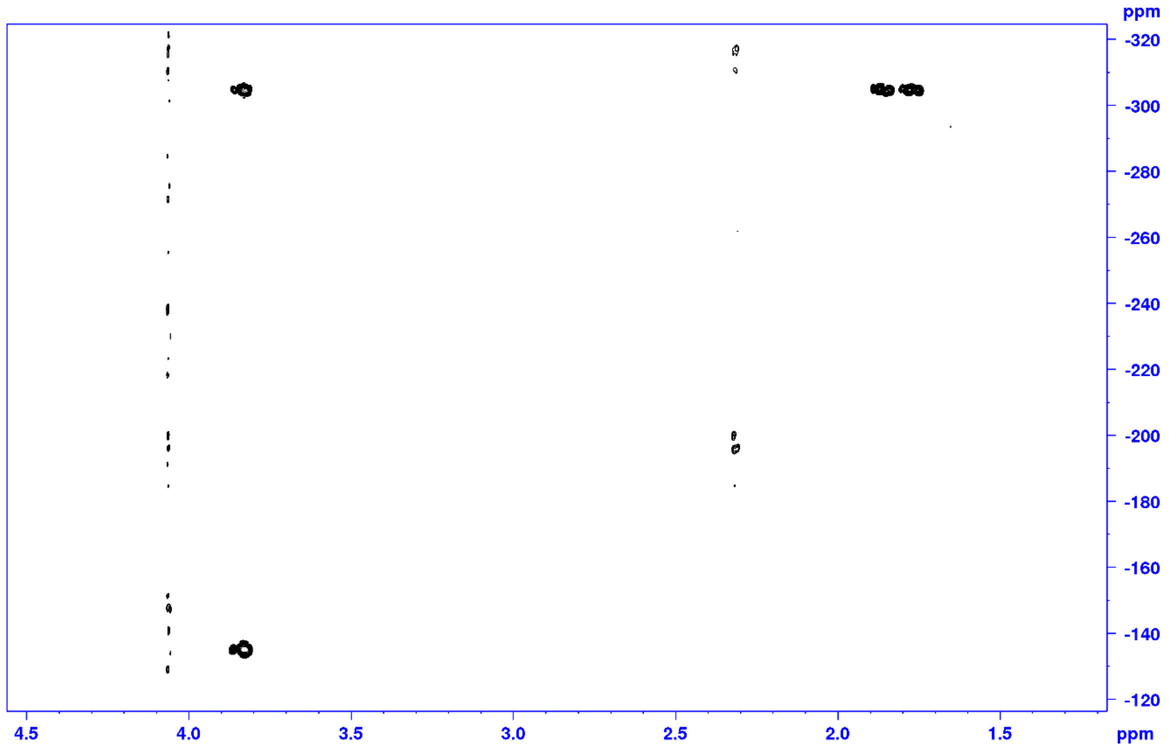

Figure S10. ^{1}H-[15]N Heteronuclear Multiple Bond Correlation spectrum (700 MHz, $CDCl_3$) of poly(αN_3εCL-*co*-εCL). The protons at 3.84 ppm are adjacent to two nitrogen atoms that match the expected shift of an alkyl azide.

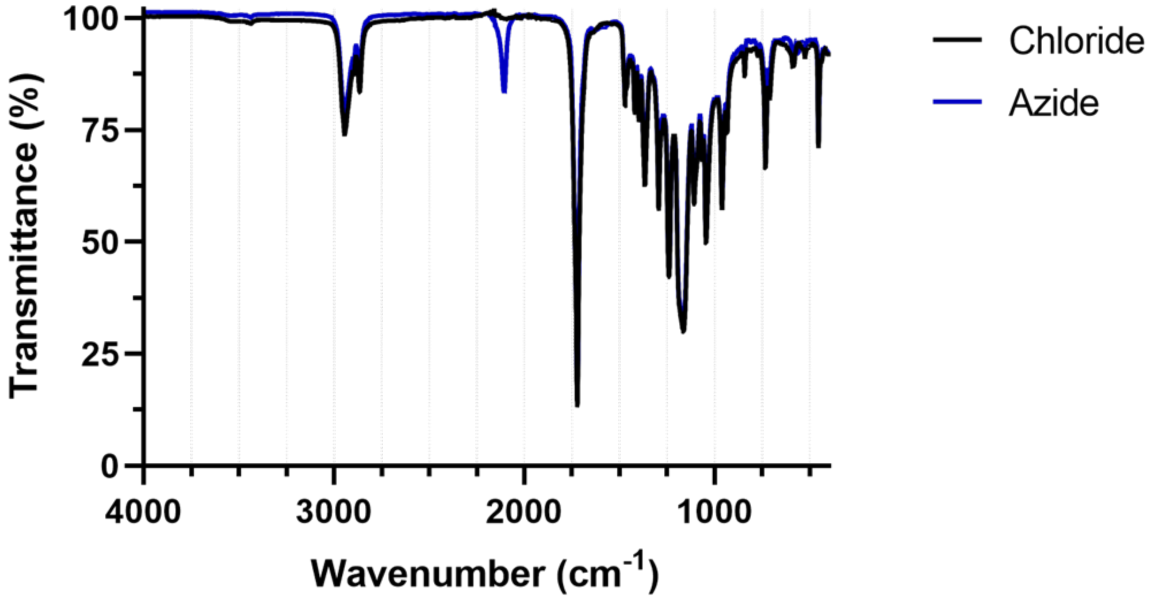

Figure S11. FT-IR spectrum of poly(αN_3εCL-*co*-εCL) (blue) and poly(αClεCL-*co*-εCL) (black). The asymmetric stretch of the azide is present at 2100 cm^{-1}

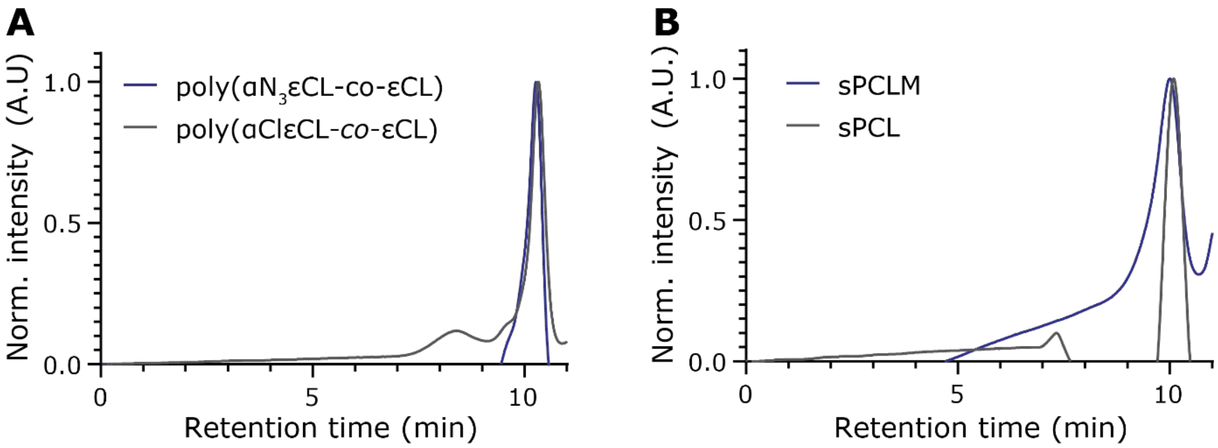

Figure S12. GPC traces of (A) poly(αN3εCL-*co*-εCL) (7.4 kg.mol^{-1}, Đ = 1.06) versus poly(αClεCL-*co*-εCL) (6.7 kg.mol^{-1}, Đ = 1.13) and (B) sPCL (9.5 kg.mol^{-1}, Đ = 1.21) versus sPCLM (13.5 kg.mol^{-1}, Đ = 1.30).

2.3 sPCLM characterization

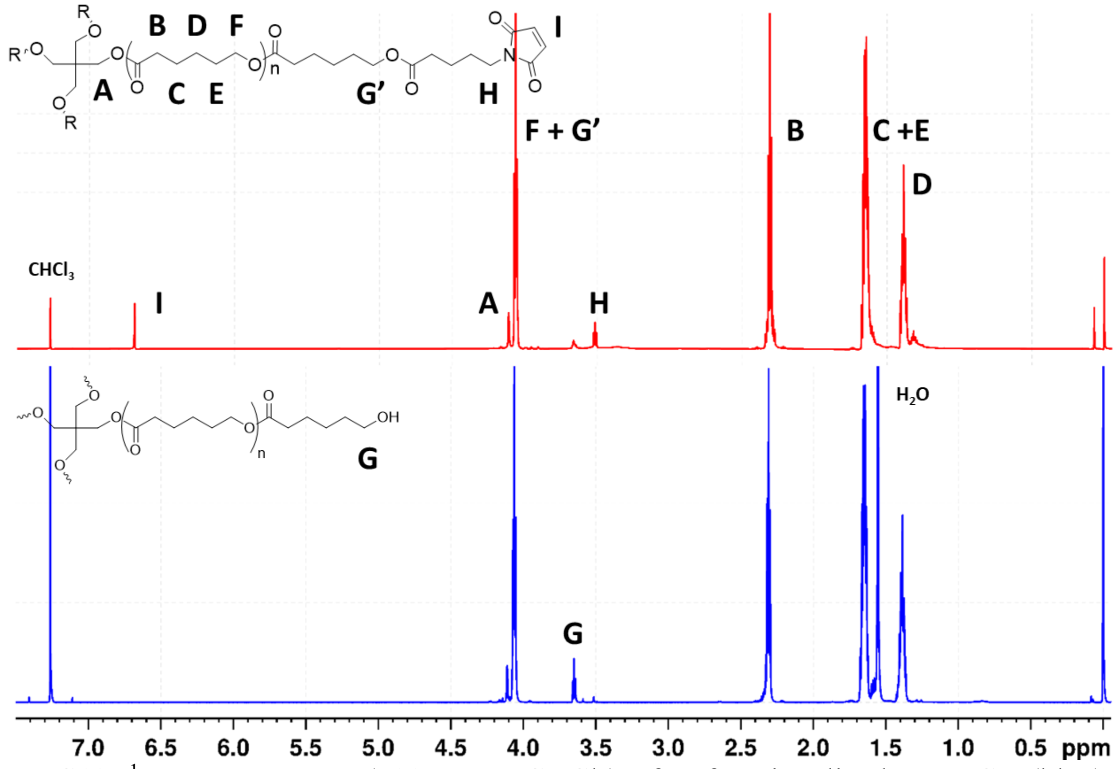

Figure S13. ^{1}H NMR spectra (700 MHz, $CDCl_3$) of unfunctionalized star-PCL (blue) and purified star-PCLM (red).

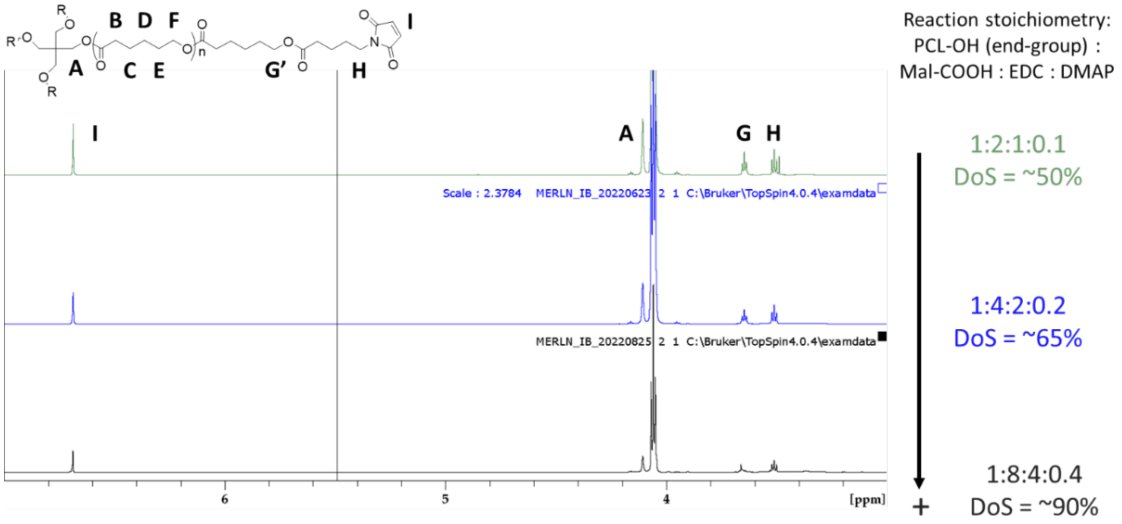

Figure S14. ^{1}H NMR spectra (700 MHz, $CDCl_3$) of purified star-PCLM after varying the stoichiometric excess of the reagents relative to the hydroxyl end group. A higher excess was added, when moving towards the bottom spectrum. The degree of substitution (DoS) was determined by setting the integral of the A protons to 2.00, and subsequent calculation of I_H / (I_G+I_H) *100. For 90% DoS, the I_G triplet was distorted. Based on the integration values of the backbone protons, it was too high. Thus, here we assumed I_H / I_A *100 for the DoS.

3. Scaffold characterization

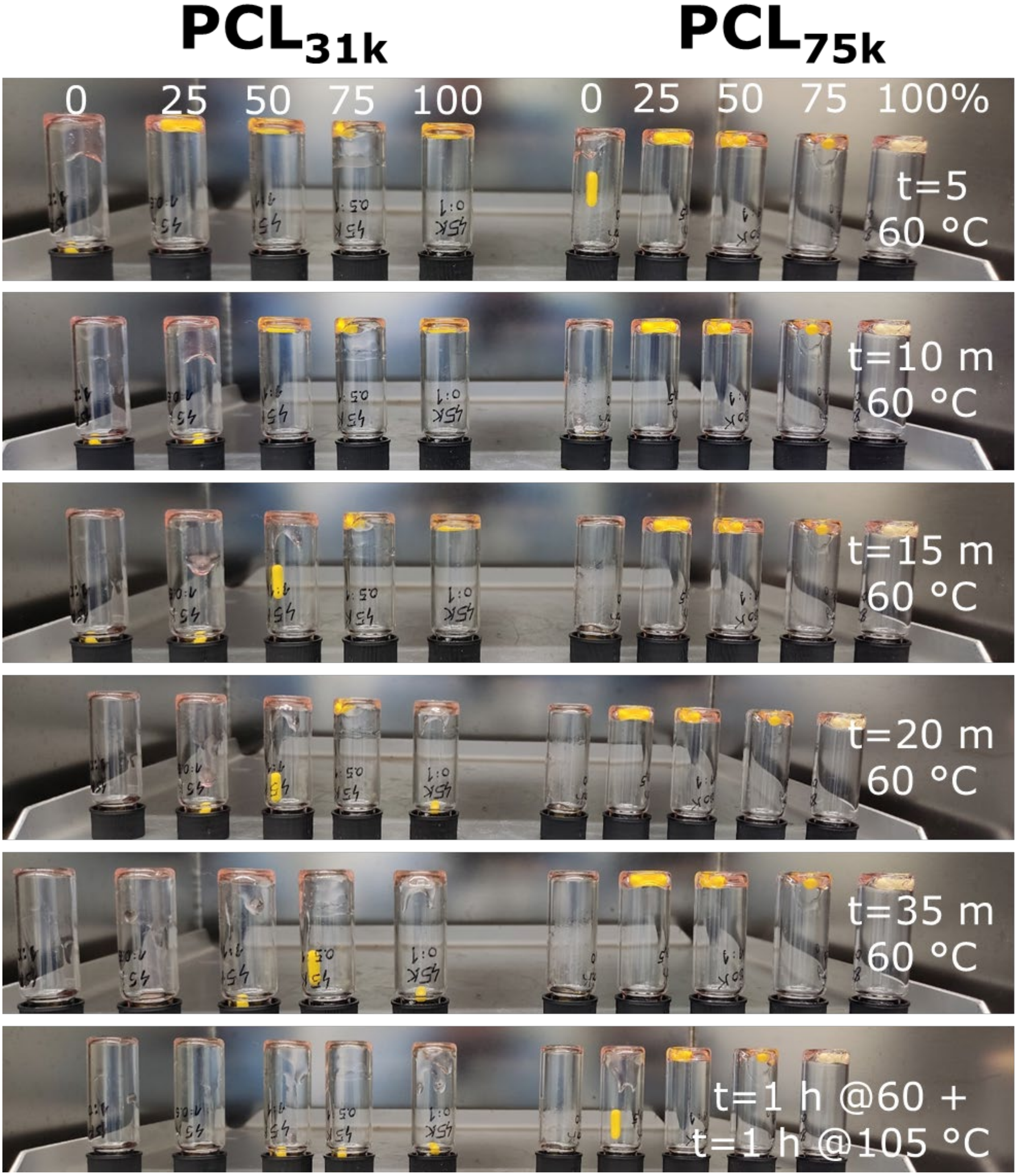

Figure S15. Qualitative assessment of the viscosity via vial inversion tests. We first melted the polymers and then inverted the vial. 0.002 wt% of hydrophobic dye and the stirrer bar were added to visualize flowing of the material.

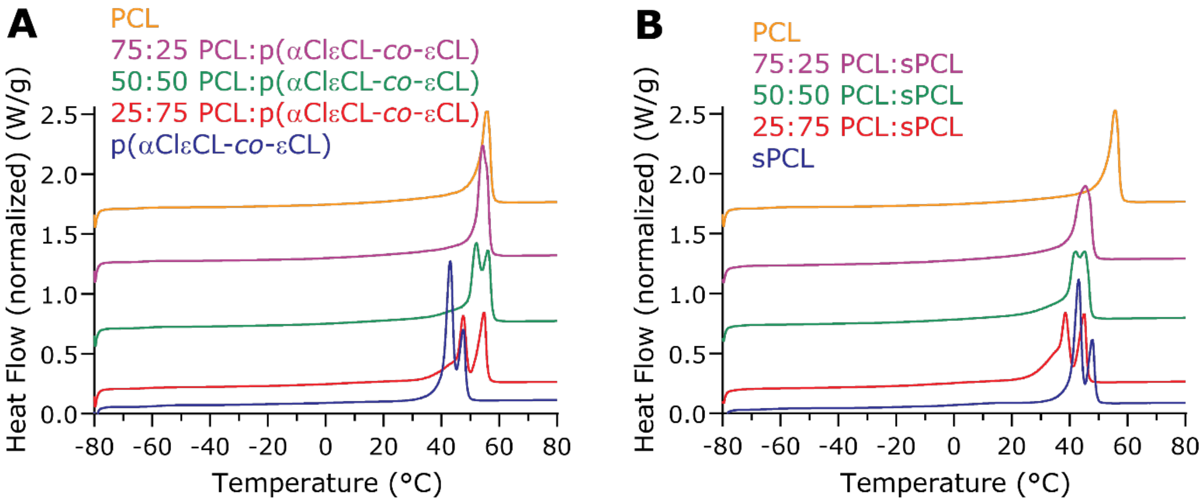

Figure S16. DSC thermogram of the second heating cycle of (A) PCL, PCL:poly(αClεCL-*co*-εCL)$_{5k}$, and poly(αClεCL-*co*-εCL)$_{5k}$ as well as (B) PCL, PCL:sPCL$_{5k}$, and sPCL$_{5k}$. Samples were heated to 100 °C, and held isothermal for 5 min to erase thermal history. Then, we cooled the samples at a rate of -5 °C.min^{-1}. In the figure, one can find the second heating cycle at 5 °C.min^{-1}.

Table S2. Melting transitions and of melting enthalpy of polymers and blends.

Blends[b] (wt:wt)	T_m[a] (°C)	ΔH_m (J.g^{-1})	T_c[b] (°C)	ΔH_c (J.g^{-1})	T_m[c] (°C)	ΔH_m (J.g^{-1})
			PCL$_{75k}$: poly(αClεCL-*co*-εCL)$_{5k}$			
100:0	59.1	63.9	25.4	51.8	55.6	53.2
75:25	58.6	71.6	28.1	60.6	54.1	58.0
50:50	58.2	77.8	25.3	62.8	x[d]	52.0
25:75	55.9	75.8	22.6	55.6	x[d]	46.2
0:100	48.9	81.7	20.6	64.9	x[d]	63.9
			PCL$_{75k}$: sPCL$_{5k}$			
100:0	59.1	63.9	25.4	51.8	55.6	53.2
75:25	61.4	70.0	24.9	58.9	55.4	50.8
50:50	59.3	72.8	25.9	63.2	x[d]	50.3
25:75	57.9	71.7	26.4	61.0	x[d]	49.8
0:100	52.1	67.7	24.1	59.7	x[d]	54.3

a) Melting transitions from the first heating cycle at a rate of 10 °C.min^{-1}.

b) Crystallization temperatures were determined at a cooling rate of 5.0 °C.min^{-1}

c) Melting transitions from the second heating run at a rate of 5.0 °C.min^{-1}.

d) A bimodal melting peak was observed.

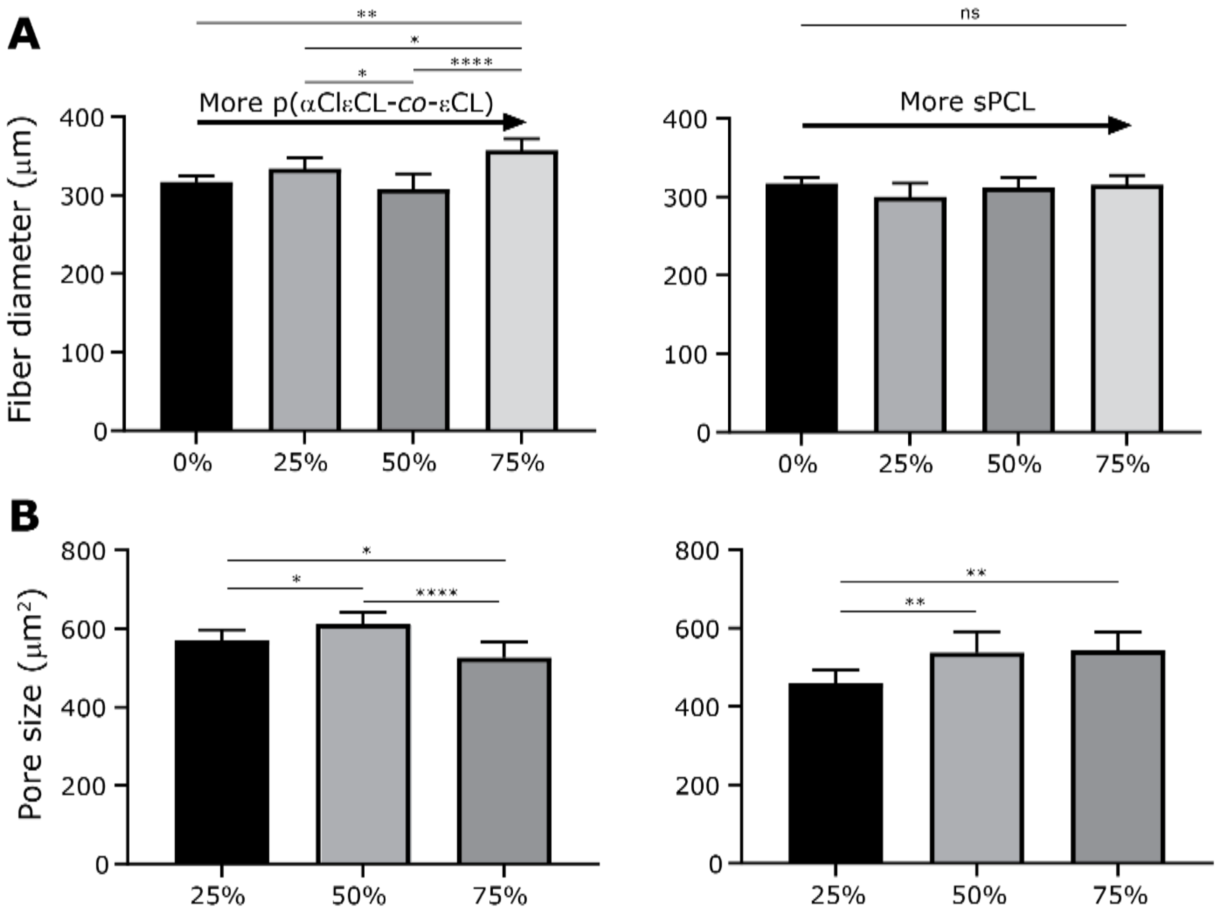

Figure S17. Fiber diameters (A) and pore size (B) of scaffolds manufactured from blends of (left) PCL_{75k}:poly(αClεCL-*co*-εCL)$_{5k}$ and (right) PCL_{75k}:$sPCL_{5k}$. N = 2. Ns = non-significant, *p<0.05, **p<0.01, ****p<0.0001.

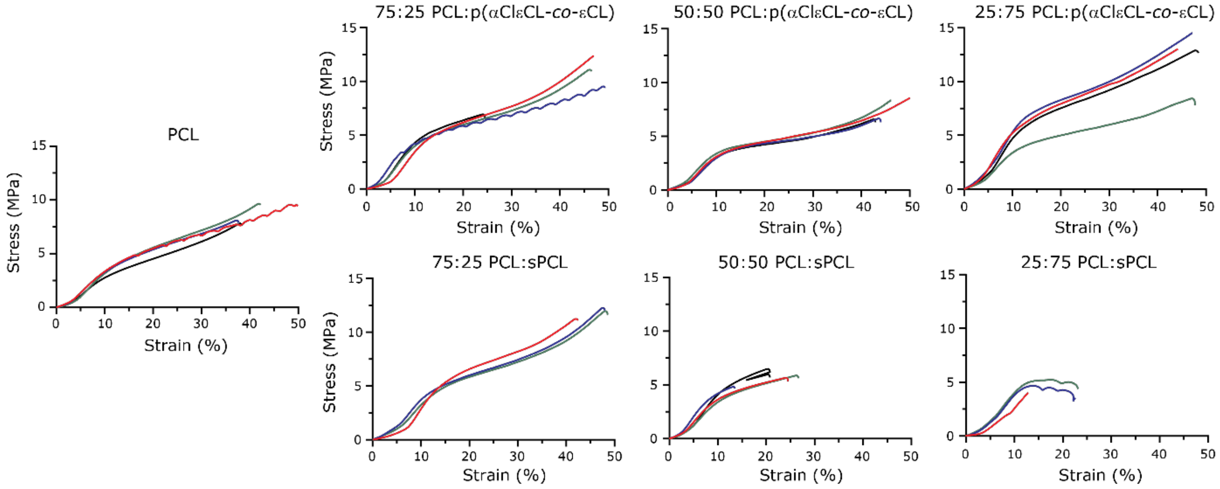

Figure S18. Compression stress strain curves of 0-90 scaffolds. The scaffolds were printed from blends comprising several weight ratios of PCL_{75k} to low molecular weight polymer ($sPCL_{5k}$ or poly(αClεCL-*co*-εCL)$_{5k}$). Representative curves were fitted in Figure 4 of the main text.

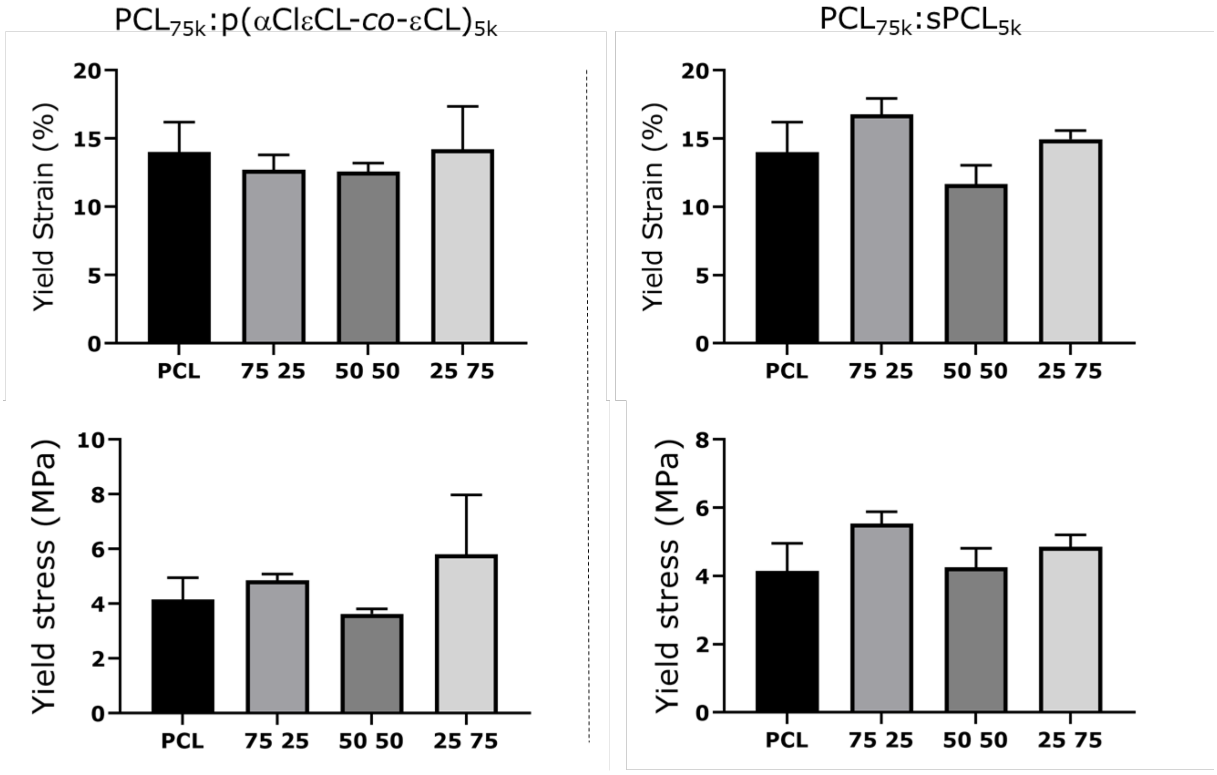

Figure S19. Yield strain (top) and yield stress (bottom), determined by 0.2% offset method. In the left and right panel, the results of the PCL_{75k}:poly(αClεCL-*co*-εCL) and PCL_{75k}:sPCL are displayed, respectively. Blend composition of a 100:0, 75:25, 50:50, and 25:75 weight ratio were used during manufacturing.

4. Characterization of surface-grafted dyes

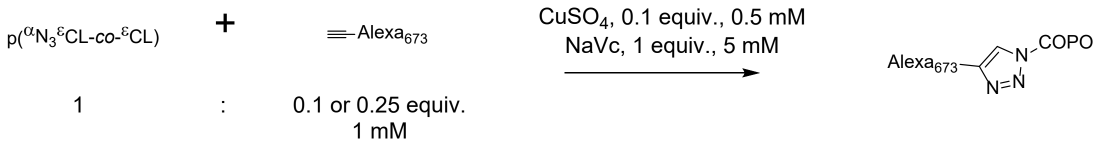

Scheme S1. Reaction of complementary fluorescent molecules on the thermopressed films using copper catalyzed alkyne azide cycloaddition chemistry. An aqueous solution containing 0.1 equiv of alkyne-Alexa673 was applied onto all films containing poly(αN3εCL-*co*-εCL)5k. On the 25:75, we also applied a solution containing 0.25 equiv of dye. The theoretical total amount of azide groups present in the films was set to 1.0 equivalent.

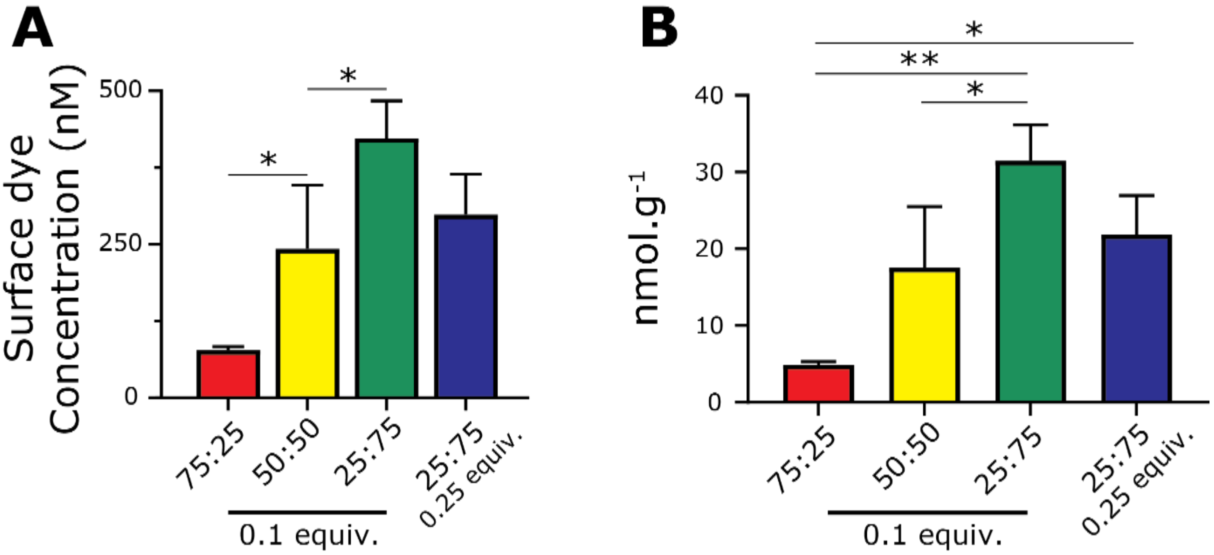

Figure S20. (A) Dye concentration after dissolving the films in chloroform, extracted from spectrafluorimetry using a standard curve. We performed the reaction on films as depicted in Scheme S1. To all films, a solution was applied containing 0.1 equiv of dye. In addition, to the surface with the highest amount of azide (25:75), we applied a solution containing 0.25 equiv of dye. (B) We converted the concentration in the solution to the amount of nmol of dye per gram of material. One could appreciate the increasing trend, aligned with the higher azide content in the film. Moreover, application of 0.25 equiv implied that saturation of the surface available azides was reached.

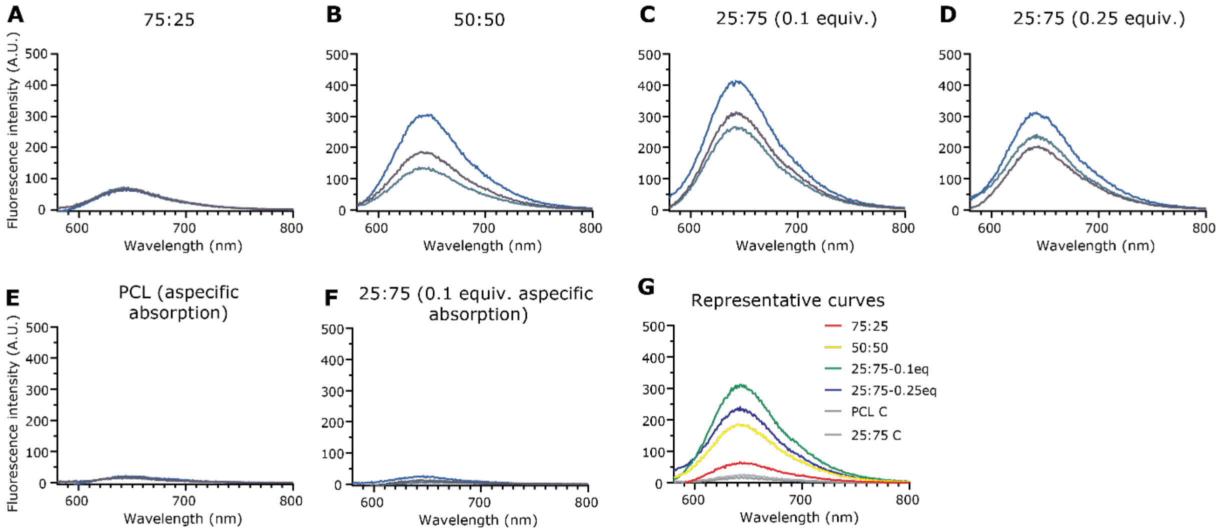

Figure S21. (A–F) All emission curves (λ_{exc} = 550 nm) of dissolved thermopressed surfaces after incubation of an alkynated dye on different compositions. In E&F, no copper or sodium ascorbate were present. (G) Representative emission curves. N=3.

5. hMSC differentiation on thermopressed films containing Poly(αN$_3$εCL-*co*-εCL)$_{5k}$ with surface-grafted BMP2 peptide

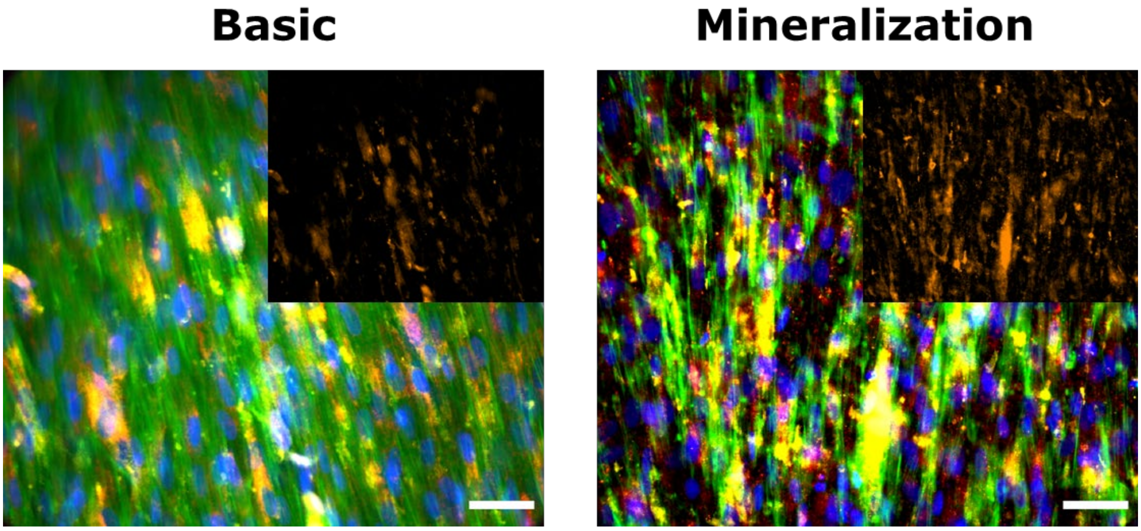

Figure S22. Immunofluorescent images of hMSCs seeded on PCL$_{75k}$ in basic and mineralization media. We stained for nuclei (blue), F-actin (green), BMPR-II (yellow), and osteocalcin (red). Scale bar is 200 µm. N=2.

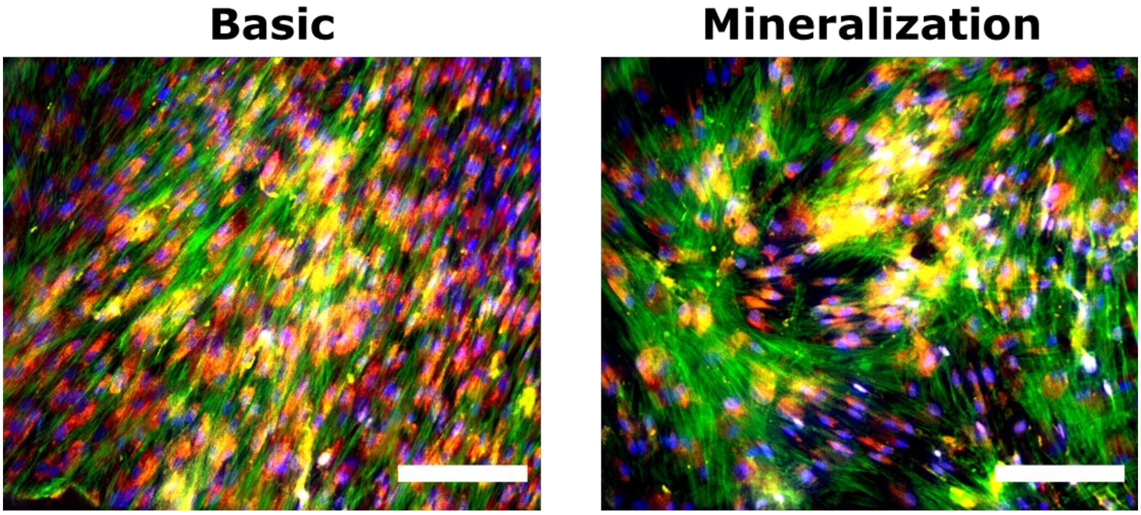

Figure S23. Immunofluorescent images of hMSCs seeded on TCP in basic and mineralization media. We stained for nuclei (blue), F-actin (green), BMPR-II (yellow), and osteocalcin (red). Scale bar is 200 µm. N=2.

6. References

[1] Beeren I A O, Dijkstra P J, Lourenço A F H, Sinha R, Gomes D B, Liu H, Bouvy N, Baker M B, Camarero-Espinosa S and Moroni L 2023 Installation of click-type functional groups enable the creation of an additive manufactured construct for the osteochondral interface *Biofabrication* **15** 014106

[2] Bolley A, Mameri S and Dagorne S 2020 Controlled and highly effective ring-opening polymerization of α-chloro-ε-caprolactone using Zn- and Al-based catalysts *J. Polym. Sci.* **58** 1197–206
